# Effects of genetic components of plant development on yield-related traits in wheat (*Triticum aestivum* L.) under stress-free conditions

**DOI:** 10.3389/fpls.2022.1070410

**Published:** 2023-02-08

**Authors:** Ádám Horváth, Tibor Kiss, Zita Berki, Ádám D. Horváth, Krisztina Balla, András Cseh, Ottó Veisz, Ildikó Karsai

**Affiliations:** ^1^ Agricultural Institute, Centre of Agriculture, Eötvös Loránd Research Network (ELKH), Martonvásár, Hungary; ^2^ Food and Wine Research Institute, Eszterházy Károly Catholic University, Eger, Hungary

**Keywords:** wheat, developmental patterns, adaptation, *PPD-D1*, minor developmental loci, multiyear field experiment

## Abstract

The dynamics of plant development not only has an impact on ecological adaptation but also contributes to the realization of genetically determined yield potentials in various environments. Dissecting the genetic determinants of plant development becomes urgent due to the global climate change, which can seriously affect and even disrupt the locally adapted developmental patterns. In order to determine the role plant developmental loci played in local adaptation and yield formation, a panel of 188 winter and facultative wheat cultivars from diverse geographic locations were characterized with the 15K Illumina Single Nucleotide Polymorphism (SNP) chip and functional markers of several plant developmental genes and included into a multiseason field experiment. Genome-wide association analyses were conducted on five consecutive developmental phases spanning from the first node appearance to full heading together with various grain yield–related parameters. The panel was balanced for the *PPD-D1* photoperiod response gene, which facilitated the analyses in the two subsets of photoperiod-insensitive and -sensitive genotypes in addition to the complete panel. *PPD-D1* was the single highest source, explaining 12.1%–19.0% of the phenotypic variation in the successive developmental phases. In addition, 21 minor developmental loci were identified, each one explaining only small portions of the variance, but, together, their effects amounted to 16.6%–50.6% of phenotypic variance. Eight loci (2A_27, 2A_727, 4A_570, 5B_315, 5B_520, 6A_26, 7A_1-(*VRN-A3*), and 7B_732) were independent of *PPD-D1*. Seven loci were only detectable in the *PPD-D1*-insensitive genetic background (1A_539, 1B_487, 2D_649, 4A_9, 5A_584-(*VRN-A1*), 5B_571-(*VRN-B1*), and 7B_3-(*VRN-B3*)), and six loci were only detectable in the sensitive background, specifically 2A_740, 2D_25, 3A_579, 3B_414, 7A_218, 7A_689, and 7B_538. The combination of *PPD-D1* insensitivity and sensitivity with the extremities of early or late alleles in the corresponding minor developmental loci resulted in significantly altered and distinct plant developmental patterns with detectable outcomes on some yield-related traits. This study examines the possible significance of the above results in ecological adaptation.

## Highlights

- A multiyear, autumn-sown field experiment was conducted to analyze plant development and yield-related traits parallel in a genome-wide association panel of 188 winter and facultative wheat cultivars from diverse geographic locations.
- *PPD-D1* had the greatest impact on plant development from early spring onward, and it influenced several morphological traits.
- There were also 21 additional minor plant developmental loci identified; most of them were *PPD-D1* allele and plant developmental phase specific.
- The combinations of the extremes of early or late alleles of minor loci with the allelic phase of *PPD-D1* resulted in significant changes in the plant developmental patterns, which altered yield parameters.

## Introduction

Plants developed complex genetic networks to control flowering time in response to the daily, seasonal, and yearly cycles of temperature and light conditions including the photoperiods characteristic to a given location. Domestication and breeding activities unconsciously modified this genetic network into such gene allele combinations that enhanced and optimized grain yield to a given region *via* plant development (Cockram et al., 2007; Trevaskis et al., 2007; Distelfeld et al., 2009; Cao et al., 2021; Fernández-Calleja et al., 2022). Human activity resulted in the evolvements of redundancy in flowering-time gene-family members, in functional allele variants, and functional polymorphisms in gene interactions, which made the geographical spreading possible (Cockram et al., 2007; Casao et al., 2011; Cao et al., 2021). Thus, the timing of plant development and the flowering of crop plants are crucial in both ecological adaptation and yield formation, and the importance of these traits increases as the disruptions in local climatic conditions and the occurrences of serious weather anomalies intensify due to climate change.

Bread wheat is considered a cosmopolitan species, and, due to its great genetic variability, it is able to adapt to a wide range of growing conditions, making its cultivation feasible in the extreme diversity of macro- and microclimates and edaphic and biotic characteristics (Cockram et al., 2007; Trevaskis et al., 2007; Distelfeld et al., 2009; Dreisigacker et al., 2021). However, this does not apply to the level of individual growing locations where only relatively few sets of wheat cultivars can be grown successfully (Karsai et al., 2012; Kiss et al., 2014; Kiss et al., 2019; Benaouda et al., 2022). The two basic features of plant development are vernalization requirement (*VRN* genes) and photoperiod sensitivity (*PPD* genes), which determine the suitability of a wheat genotype for the different wheat-growing megaenvironments (Worland et al., 1998; Cockram et al., 2007; Trevaskis et al., 2007; Distelfeld et al., 2009; Cao et al., 2021). None of these megaenvironments, however, is homogenous, but they represent rather rich combinations of various environmental factors (Benaouda et al., 2022). The meteorological and climatic parameters of the location–especially the maximum temperatures in spring–play the most decisive roles in influencing plant development (Jung et al., 2021; Benaouda et al., 2022). Thus, in addition to the two basic features, the determination of plant developmental patterns includes the sets of interconnected earliness per se genes that together ensure the fine-tuning of ecological adaptation (Cockram et al., 2007; Griffiths et al., 2009; Dreisigacker et al., 2021).

In cereals, the vernalization requirement of a genotype is determined by the epistatic interactions between two major vernalization response gene families, *VRN1* and *VRN2*. *VRN1* loci encode dominant flowering activators, while *VRN2* loci encode dominant repressors of flowering (Trevaskis et al., 2003; Yan et al., 2003; Yan et al., 2004; Fu et al., 2005; Karsai et al., 2005; von Zitzewitz et al., 2005; Faure et al., 2007; Trevaskis et al., 2007; Díaz et al., 2012). Depending on the ratio of dominant and recessive alleles in the *VRN* genes in the three genomes of hexaploid wheat, it is possible to distinguish cultivars with winter or spring seasonal growth, while genotypes with the facultative habit have various combinations of dominant and recessive alleles. *PPD1* is the major gene of photoperiod sensitivity (Turner et al., 2005; Beales et al., 2007; Bentley et al., 2013; Langer et al., 2014), and it acts with both the photoperiod regulating pathway and the circadian clock. In wheat, the dominant photoperiod insensitivity alleles in *PPD1* result in rapid heading irrespective of the photoperiod that is more pronounced under short photoperiods. *VRN3* genes (*TaFT1*) are the central integrators of the vernalization and photoperiod pathways, and, as such, they are under the control of *VRN1*, *VRN2*, and *PPD1* series (Yan et al., 2006; Cockram et al., 2007; Campoli and von Korff, 2014). The functional polymorphisms in these plant developmental genes have been thoroughly analyzed (Guo et al., 2010; Nishida et al., 2013; Berezhnaya et al., 2021). SNPs, various sizes of Insertion-deletion mutation (INDELs), transposon insertions in the different parts of the developmental genes, and copy number variations (CNVs) resulted in several functional allele variants, but, for each gene, one distinct variation defines its role (Beales et al., 2007; Zhang et al., 2008; Guo et al., 2010; Díaz et al., 2012; Nishida et al., 2013; Kiss et al., 2014). In the case of the *VRN1* locus, a large deletion in intron1 results in the dominant spring allele, while for *VRN2* locus, a point mutation in the CCT region or the complete absence of the *ZCCT* genes results in the recessive spring allele (Yan et al., 2003; Yan et al., 2004; Fu et al., 2005). For the *PPD1* locus, however, the basis for insensitivity is species and gene specific (Turner et al., 2005; Beales et al., 2007; Langer et al., 2014; Fernández-Calleja et al., 2022). In bread wheat, the insensitive *PPD-B1* allele harbors increased copy numbers of the gene, while, for *PPD‐D1*, it is a large deletion of 2,089 bp in the promoter region leading to overexpression and an altered circadian rhythm (Beales et al., 2007).

When the vernalization and photoperiod requirements are saturated resulting in optimal conditions to flower induction, the third class of genes, namely, those of *eps*, play an important role in fine-tuning the plant development (Valárik et al., 2006; Griffiths et al., 2009; Langer et al., 2014; Basavaraddi et al., 2021). The underlining genes and causal polymorphisms at the various *eps* loci have recently been identified in cereals (Campoli et al., 2013; Zikhali et al., 2014; Sukumaran et al., 2016; Zikhali et al., 2017). As the allele phases in *VRN1* and *PPD1* in cereals determine the basic plant developmental characteristics, the possible functions of the less effective flowering time genes depend strongly on their complex epistatic interactions, which can modify their expressions and mode of actions (Mulki and von Korff, 2016; Basavaraddi et al., 2021; Cao et al., 2021; Fernández-Calleja et al., 2022). To illustrate this phenomenon, the overexpressions of the cereal orthologs of *CO*, the central element of the *Arabidopsis* photoperiodic pathway, may delay or accelerate flowering in cereals depending on the allele phases in *PPD1* and/or *VRN1* (Mulki and von Korff, 2016; Shaw et al., 2020). This may account for the difficulty to identify the various *eps* genes; their effects may only be detected at specific *VRN1*–*PPD1* allele structures.

The lengths of the consecutive phenological stages and their transfer from one stage to another affect various grain yield–related traits (González et al., 2005a; Gonzalez-Navarro et al., 2016; Guo et al., 2018a; Kiss et al., 2019). Extending the duration of phase intervals that impact yield components without modifying the total time to anthesis has been proposed as a promising breeding tool. This approach, however, requires an in-depth knowledge of the influence of plant developmental genes on yield formation (González et al., 2005a; González et al., 2005b; Borrás-Gelonch et al., 2012; Gonzalez-Navarro et al., 2016; Prieto et al., 2018). Under field conditions, however, the various combinations of environmental factors experienced in different locations and years result in a considerable variability in the phenotypic effects of the individual alleles, often leading to contradictory findings (Snape et al., 1985; Worland et al., 1998; Kato et al., 2000; Gonzalez-Navarro et al., 2016; Prieto et al., 2018). The direct impacts of these genes on the yield can mostly be identified when the contrasting functional alleles of the *VRN1* and *PPD1* genes are compared or the same wheat germplasms are grown under different and contrasting environmental conditions (González et al., 2005a; Dreisigacker et al., 2021; Benaouda et al., 2022). In the latter case, the difference in the level of various abiotic stresses may also confound the exact dissection of the associations. It has been established that allelic differences and their combinations in vernalization response (*VRN*) and photoperiod sensitivity (*PPD*) genes impact abiotic stress responses in temperate cereals (Voss-Fels et al., 2018; Alipour and Abdi, 2021; Abu-Elenein et al., 2021; Gol et al., 2021; Ochagavía et al., 2022). However, very little is known about how the genes that synchronize the developmental phases affect the actual formation of grain yield within a growing environment. To uncover the key roles of flowering genes in local adaptation and yield formation, it is important to characterize the existing genetic variation for plant phenology in diverse collections of wheat landraces and cultivars. This can assist breeding programs to improve resilience and adaptation to local environments.

In order to dissect the genetic determinants of plant development and determine their role in local adaptation and yield formation, a set of 188 winter and facultative wheat cultivars from various geographic locations were characterized with the 15K Illumina SNP chip and included in a multiseason field experiment (Kiss et al., 2019). GWA analyses were conducted on five successive developmental phases spanning from the first node appearance to full heading combined with various grain yield–related parameters. The specificity of the wheat panel meant that the lines carried the *PPD-D1*-sensitive or -insensitive alleles in equal proportion. This scheme facilitated the identification of minor plant developmental loci that are independent of the *PPD-D1* gene as their effects may be directly detected in the entire population. In addition, the detection of minor developmental loci showing specific associations with the allele phase of *PPD-D1* was also feasible. The application of the combined scheme of *PPD-D1* and these minor loci revealed the genetic bases of the phenotypic variations in plant developmental parameters and demonstrated the extent of their contributions to yield determinations.

## Materials and methods

### Plant material

A total of 188 winter and facultative wheat accessions from various geographic locations were used for this GWA study. Information on the cultivars and their origins has been published by Kiss et al. (2019), and it is available for further requests. They were selected from an initial wheat population consisting of 683 wheat genotypes from the GeneBank collection of the ELKH ATK MGI (Agricultural Institute, Centre of Agriculture, Eötvös Loránd Research Network, Martonvásár, Hungary), which had been previously examined for heading date characteristics and allele types in the major plant developmental genes (Kiss et al., 2014). Selection was primarily based on the *PPD-D1* photoperiod sensitivity allele type; thus, within this group of 188 genotypes cca., one-half carried the insensitivity and the other half carried the sensitivity allele. In addition, geographical and heading date heterogeneity within each pool was maintained. The wheat genotypes originated from 24 countries (present or former locations) that could be grouped into six larger geographic regions. These were (1) West Europe (n = 35; A, CH, D, F, and GB); (2) Central Europe (n = 71; CZ, SK, H, and HR); (3) East Europe (n = 9; R, RUS, and UKR); (4) Southern Europe (n = 16, I, MK, and former YU); (5) America and others (n = 33; CAN, USA, ARG, AUS, and RSA); and (6) Asia (n = 24; TR, IRN, CN, and J).

### Phenotypic evaluations

#### Multi-year, one location experiment

The field experiments were conducted in three consecutive years (2013 – 2015) at the same location, in the Centre for Agricultural Research, ELKH, Martonvásár, Hungary (Latitude: 47° 21’ N, Longitude: 18° 49’ E, Altitude: 150 m) to allow the comparison of seasonal effects. In each year, the sowing were implemented around 10 October using the same experimental design. 186 wheat genotypes were sown without replications, while two (the early heading cultivar ‛Mv Toborzó’ and the medium-late heading cultivar ‛Mv Verbunkos’) were sown in seven replications evenly spaced across the experimental field as controls. Two rows of each genotype were sown in a 0.4 × 2 m plot with the distance of 20 cm. The specifics of the experimental set-up, the plant management, and the meteorological characteristics of the three growing seasons are published by Kiss et al. (2019). Various developmental, morphological, and yield-related traits were measured and analyzed in details and published in Kiss et al. (2019), including two-way ANOVAs. Genome-wide association studies (GWA) were conducted for several traits within each group. Plant developmental traits included the effective thermal times (ETTs; Bogard et al., 2015) collected from sowing to the start of intensive stem elongation (Z30, on the Zadoks’ scale; Tottman and Makepeace, 1979), to first node appearance (Z31), to booting stage (Z49), to full heading (Z59), and to the end of intensive stem elongation (ZSE). The ETT is used to normalize the seasonal effects. It is the total of the daily average thermal time, which is complemented by the value that indicates the length of daylight and the saturation level of the average vernalization requirement of the plants. In the equation ETT = ∑(TT × FV × FP), TT is the daily average thermal time, FV is the vernalization factor (being 0 before the saturation of the vernalization requirement), and FP is the photoperiod factor (being < 1 under a 12-h photoperiod, proportional to the actual day length). More information on the calculation of effective thermal time is published in Bogard et al. (2015). At full maturity, six plants per plot were harvested to establish various morphological and grain-yield related parameters (Kiss et al., 2019). From the group of morphological traits, plant height from soil to the base of the main spike (PH), length of the last internode (LIN), length of the main spike (EaL), number of spikelets per main spike (SPIK), and the rate of the intensive stem elongation (ETT/cm; SG) were included in the GWA. From the group of yield-related traits, GWAs were conducted for seed number per spikelet on the main spike (SSP), for the seed number (MS) and weight (MSW) on the main spike. In addition, GWA was also run on average seed number (AS), average seed weight (ASW), average thousand kernel weight (AET), and grain yield per plant (GY). The results of the two-way ANOVAs of these traits are presented in **Supplementary Table 1**.

### Genotypic characterization

Genomic DNA extractions—according to the manufacturer’s instructions—were conducted from fresh leaf samples (100 mg) using the DNeasy^®^ Plant Mini Kit (Qiagen GmbH, Hilden, Germany). The genotypes were described with various marker systems, including the sets of plant developmental gene specific primers (*PPD-B1*, *PPD-D1*, *VRN-A1*, *VRN-B1*, *VRN-D1*, *FT3-1A*, *FT3-1B*, *Rht-B1*, and *Rht-D1*, 1B/1R translocation; Kiss et al., 2014). In the cases of *VRN-A1* and *PPD-B1*, the CNV was also determined *via* the multiplex TaqMan^®^ assay (IDna Genetics Ltd., Norwich, UK). Diversity Arrays Technology (DArT) analysis was performed by Diversity Arrays Technology Pty Ltd. (CSIRO, Yarralumla, ACT, Australia; Kiss et al., 2021), 15K Infinium analysis was performed by the TraitGenetics GmbH (Gatersleben, Germany), and the SNPs represent a subset of markers from the Illumina wheat 90K SNP array (Wang et al., 2014).

### Population structure analysis and association mapping

After filtering the various markers for missing values <10% and minor allele frequency >0.05, 7,273 SNPs and gene-specific markers with known physical positions were retained for further marker analyses. The population structure and the genetic relatedness were established by two different approaches. In the first case, 249 perfect DArT markers, covering the genome evenly, were used to determine the population structure with the help of the STRUCTURE 2.3.4 program (Pritchard et al., 2000). The results of these analyses had been published earlier by Kiss et al. (2021). In that approach, the presence of four subpopulations was identified (**Supplementary Figure 1A**), which corresponded with the geographic locations (Kiss et al., 2021).

The high-quality SNP calls for 188 wheat lines were combined to construct a matrix, and a neighbor-joining-based (Saitou and Nei, 1987) unrooted dendrogram was produced using the Genome Association and Prediction Integrated Tool (GAPIT 3.0) package in R (Wang and Zhang, 2021). Principal component and kinship analyses were conducted using GAPIT with default parameters. A kinship heat map was used to analyze the genetic correlation among 188 diverse wheat lines. Principal components applied to the genotypes provide information about population structure.

To complete association mapping for our genome-wide association study (GWAS) analyses, we used 7,273 polymorphic SNPs, which had physical positions based on IWGSC (The International Wheat Genome Sequencing Consortium) RefSeq v1.0 (Alaux et al., 2018). GWAS was conducted by the compressed mixed linear model using the GAPIT package (Lipka et al., 2012). The correction for population stratification and cryptic relatedness was performed by employing kinship randomly to minimize false positives (Kang et al., 2008). The appropriateness of the model used for association analysis in the present study was tested by drawing a QQ (quantile-quantile) plot between expected and observed -log10(P) values. The significance threshold was set by an application of an overall cut-off significance level of −log10 (P-value) ≥ 3.0, where one false positive was expected within 1,000 events (Zanke et al., 2015; Jung et al., 2021). The QQ- and circular-Manhattan plots were drawn by the CMplot package in R (Yin et al., 2021). When more than two significant SNPs were identified for a given trait, multiple regression analyses were conducted additionally, with the STATISTICA software package, version 13.5.0.17 (TIBCO Software Inc.) in order to further clarify the weights of the significant SNPs and the possible associations between them. The same software package was used to conduct further exploratory analyses including K-mean clustering, discriminant analysis, and principal component analyses for various grouping purposes of the genotypes.

## Results

### Genetic diversity and population structure of the hexaploid wheat panel

**Figure 1:**
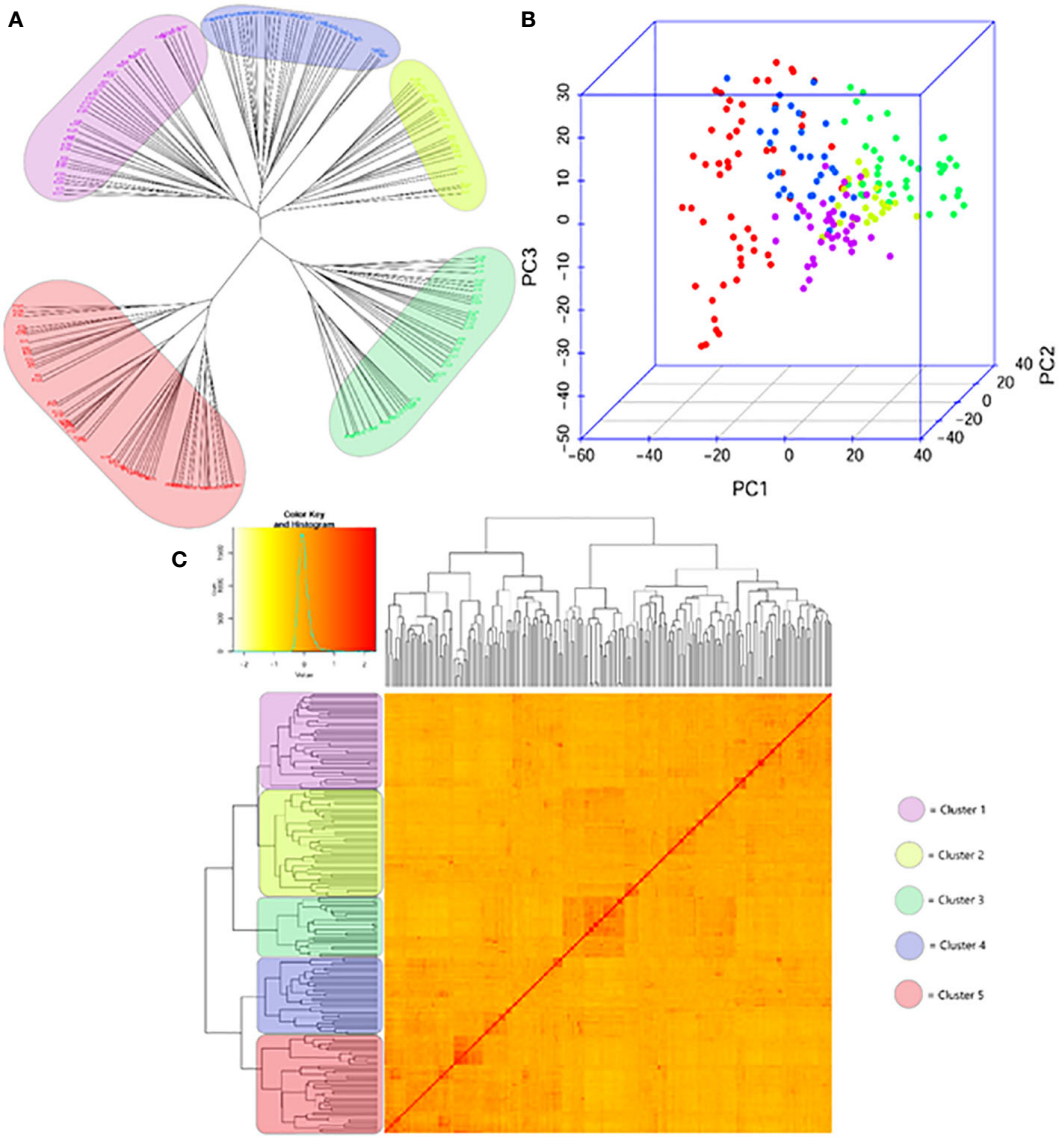
Genetic diversity and population structure of the genome wide association panel consisting of 188 winter and facultative wheat cultivars of diverse geographic origins; **(A)** NJtree, **(B)** PCA plot and **(C)** KS matrix. The colour scheme is as follows: Cluster 1 – pink; Cluster 2 – yellow, Cluster 3 – green, Cluster 4 – blue and Cluster 5 – red.

Using the matrix of 7,237 SNPs and gene-specific markers to establish the genetic relatedness of 188 wheat genotypes, a structured population was identified; the genotypes were positioned in five distinctly separated subpopulations (**Figure 1**). However, differences were observed in the level of relatedness within the individual subpopulations, and even within-group diversity was observed in Clusters 1 (pink), 2 (yellow), and 4 (blue), while Cluster 5 (red) and especially 3 (green) showed further strong inner structures. A relatively fair overlap was apparent when we compared the population structures obtained *via* the selected ideal 247 DArTs evenly distributed across the genome (Kiss et al., 2021) to the structures obtained based on the whole data sets of 7,237 SNPs (**Supplementary Figure 1B**). Applying the latter approach, however, showed more sensitivity to detect additional relatedness, thus further fine-tuning the population structure as it better clarified the positions of genotypes transiently positioned at the edges of the first three major clusters of the former approach.

A stronger association between the subpopulations and geographic locations was observed (**Supplementary Figure 1C**). Considering the geographic location most frequently associated with in each subpopulation, 63.4% of Cluster 1 came from America (in addition, from Australia and Africa), 73.1% of Cluster 3 was of West-European origin, while the Central-European cultivars were the most frequent in Cluster 4 (45.5%) and 5 (76.2%). Cluster 2 was the most mixed group including cultivars from most megaregions to a similar ratio with the exception of East Europe. East-European cultivars were distributed between Clusters 4 and 5. South-European and Asian genotypes were present in most of the clusters with the only exception of Cluster 3 with the West-European majority. Central-European wheat cultivars were present in all the clusters to varying extents. This and the special subgroups within each cluster closely linking Central-European cultivars with any members of other geographic locations are the result of the widespread practices of Central-European breeding programs, i.e., the use of crossing partners from different regions outside Central Europe (Karsai et al., 2012).

**Figure 2:**
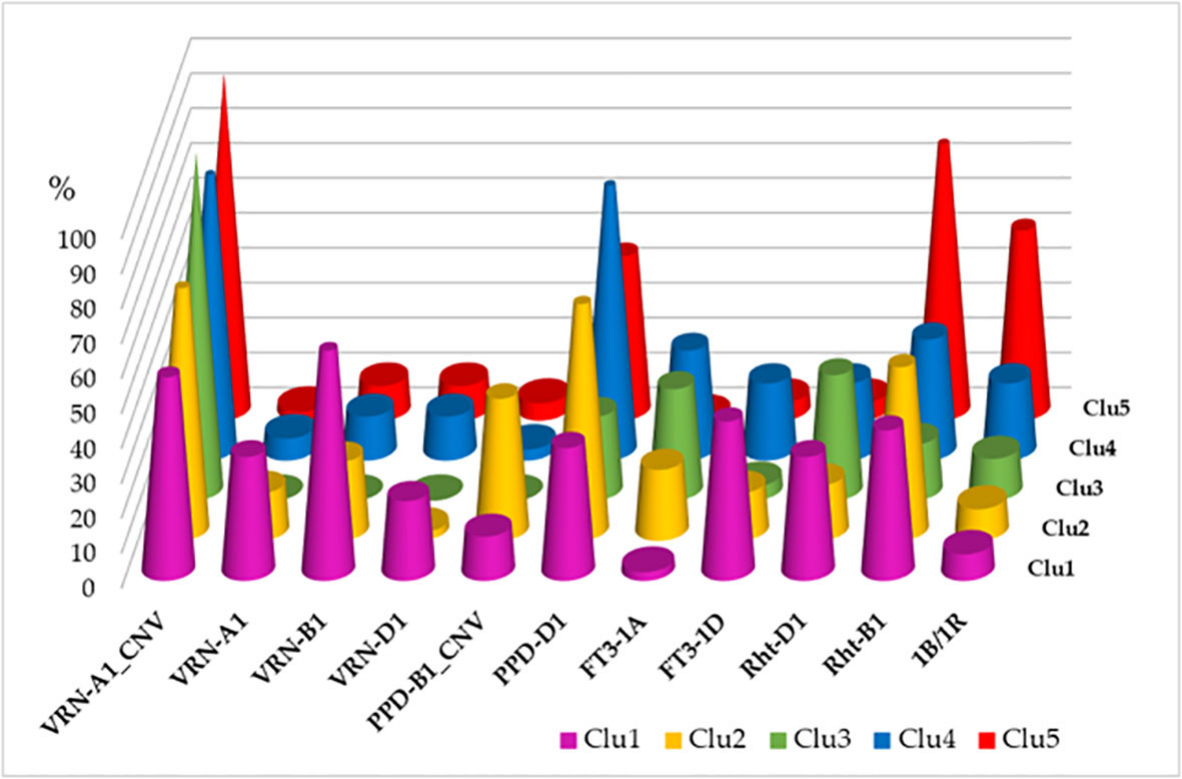
Distributions of the plant developmental gene alleles across the five genotypic clusters of 188 wheat cultivars (more than 1 copy in *VRN-A1*, spring alleles in *VRN-A1*, *VRN-B1* and *VRN-D1*, more than 2 copies in *PPD-B1*, insensitivity allele in *PPD-D1*, early allele types in *FT3-1A* and *FT3-1D*; dwarf alleles in *Rht-B1* and *Rht-D1*, presence of the 1B/1R translocation).

The five genotypic clusters showed differences in the allele frequencies of the various plant developmental genes (**Figure 2**). Clu1 was the most diverse as it carried the spring alleles in the three *VRN1* genes with the highest frequencies, and it had an early allele type in *FT3-1D* (Zikhali et al., 2014). The more frequent presence of the insensitive alleles in both *PPD-B1* and *PPD-D1* genes was characteristic to Clu2, while Clu3 was the most distinct in having no spring alleles at all in the three *VRN1* genes, with the highest ratio of CNV in *VRN-A1* and the lowest ratio of the *PPD-D1*-insensitive allele. Clu4 was the most different in having the highest number of *PPD-D1* insensitive allele, while Clu5 carried the mutant *Rht-B1* gene with the highest frequency, in addition to the high ratio of CNV in *VRN-A1*.

### Genetic components of plant development in the group of 188 winter wheat cultivars

Two-way ANOVAs were carried out on the various phenotypic traits, and both main factors—genotype and year—were highly significant components of trait variances (**Supplementary Table 1**). Genotype as a main factor explained between 41.3% (ZDSE) and 60.0% (ZD59) of the variance in the developmental parameters. Similar were the cases for the morphological and yield-related traits, justifying the appropriateness of GWA analyses.

**Figure 3:**
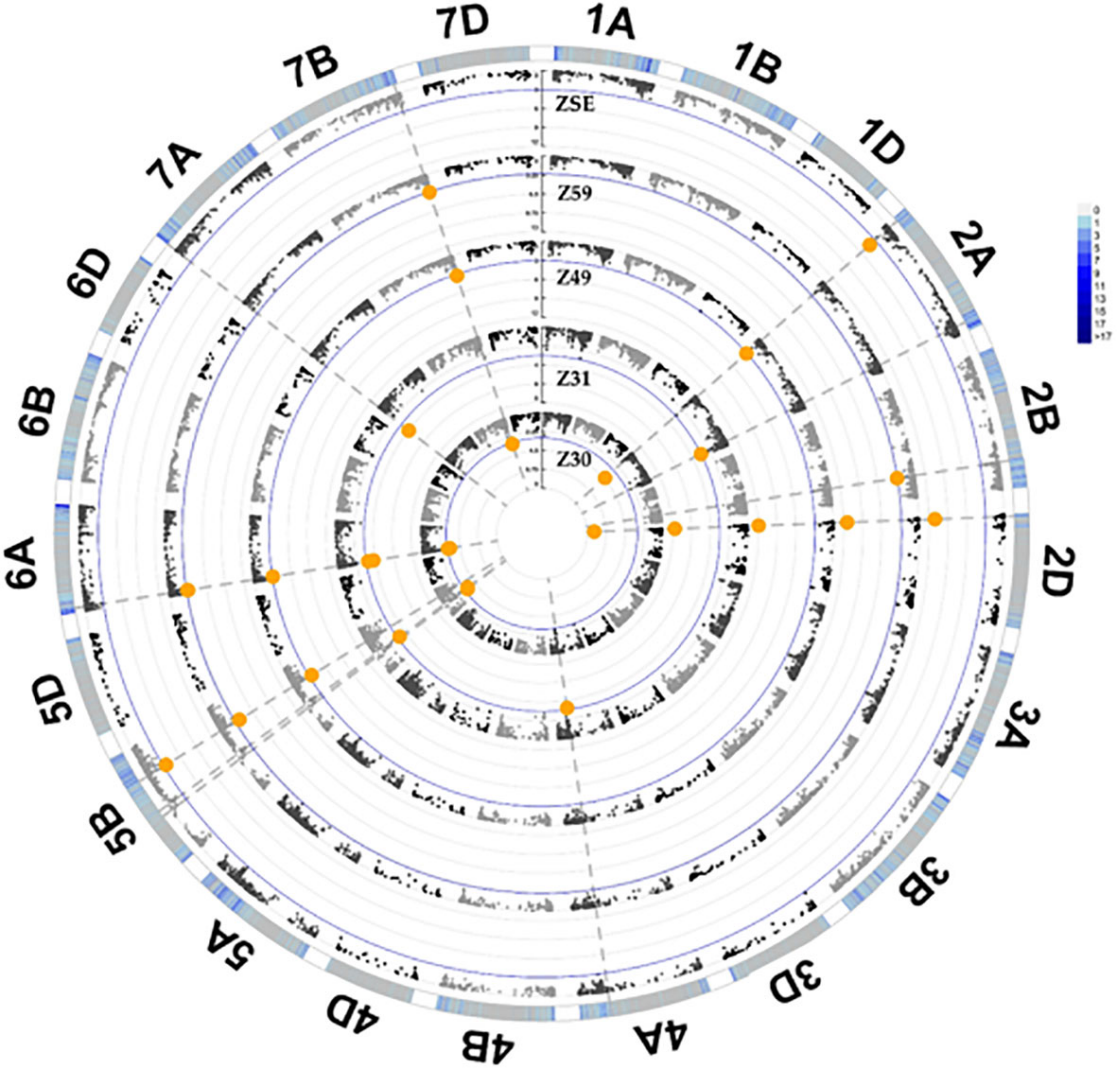
Circular Manhattan Plots of five consecutive developmental stages in the group of 188 winter and facultative wheat genotypes; Z30 – start of the intensive stem elongation; Z31 – first node detectable; Z49 – booting; Z59 – heading; ZSE – end of intensive stem elongation. The yellow dots are indicating the most significant marker-trait associations.

**Figure 4:**
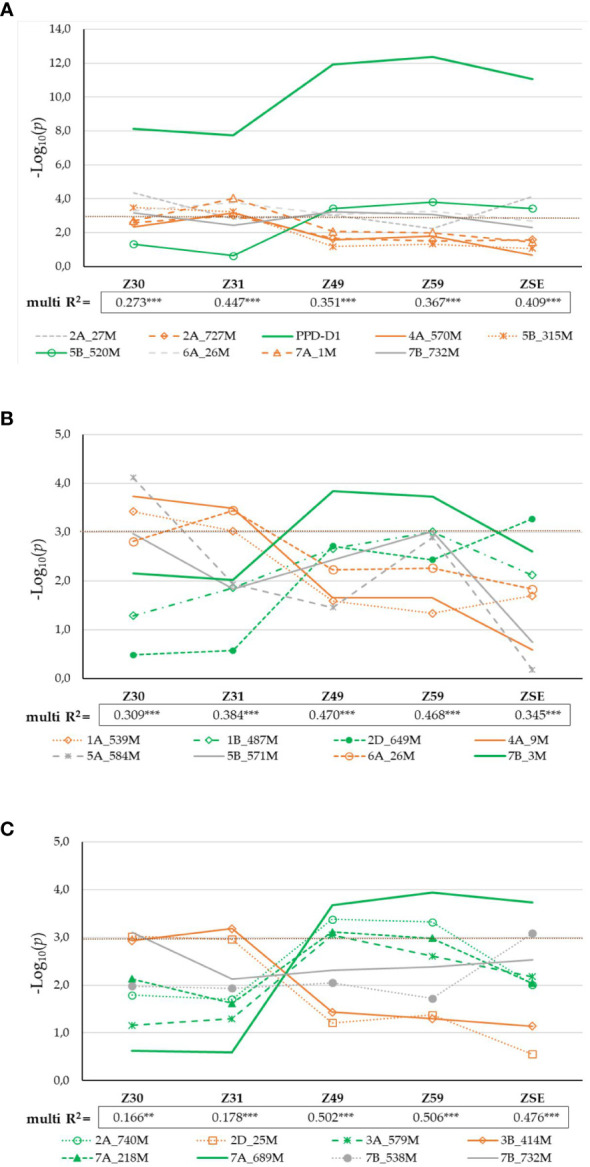
Significance profiles of markers across the consecutive developmental phases **(A)** in 188 winter wheat genotypes; **(B)** in 95 *PPD-D1* insensitive wheat genotypes; and **(C)** in 93 *PPD-D1* sensitive wheat genotypes. The LOD values originate from the GWA.

**Figure 5:**
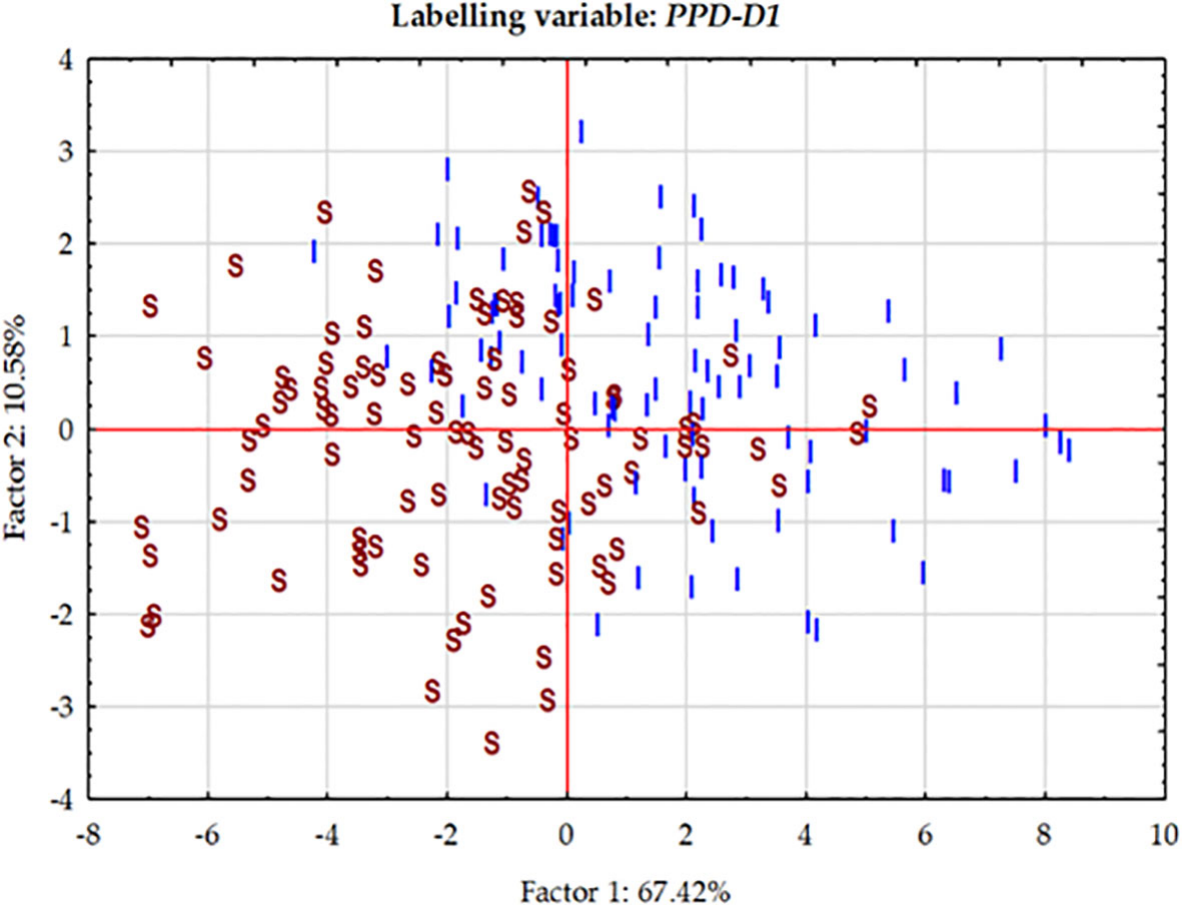
Principal Component analysis carried out on the phenotypic data matrix of the effective thermal times of five consecutive developmental phases across three seasons in the group of 188 winter wheat genotypes. The cases are labelled by the allele phase in the *PPD-D1* gene (I stands for insensitive, S for sensitive).

The numbers of marker–trait associations (MTAs) identified for the five consecutive developmental phases varied between three (ZSE) and nine (Z30), with partially overlapping SNPs (**Figure 3**; **Supplementary Table 2**). After the exclusions of markers with complete linkage to any others, nine significant SNPs were retained on seven different chromosomes (2A, 2D, 4A, 5B, 6A, 7A, and 7B; for more information on the SNP markers, see **Supplementary Table 6**). The correlation between these SNPs was in the range of 0.00 and 0.47 in the absolute value. Visualizing the effects of these SNPs *via* their LOD score changes across the consecutive developmental phases; two important aspects can be highlighted (**Figure 4A**). One is the decisive significance of the photoperiod sensitivity gene, *PPD-D1* on 2D on all the developmental phases. Its contribution increased with the later developmental phases, reaching its plateau at Z49 and Z59, with the LOD scores of 11.9 and 12.4, explaining 15.5% and 16.1% of the phenotypic variations, respectively (**Supplementary Table 2**; **Figure 4A**). Based on their developmental patterns, the wheat genotypes carrying the insensitive (I) or the sensitive (S) alleles formed two partially overlapping groups underlining the basic defining role of *PPD-D1* (**Figure 5**). In addition, the remaining eight SNPs can also be characterized with developmental specific significance patterns. Three SNPs, 2A_27M (where M refers to million base pairs), 6A_26M, and 7B_732M, influenced several phases throughout the development. The early plant development, especially Z31, was determined by four SNPs; 2A_727M, 4A_570M, 5B_315M, and 7A_1M, while 5B_520M was a significant component of Z49 and Z59, showing a pattern very similar to that of *PPD-D1*, although with a much less significance (r = 0.14 existed between *PPD-D1* and 5B_520M). Based on multiregression analyses, these SNPs together explained between 27.3% (Z30) and 44.7% (Z31) of the phenotypic variations, all highly significant.

**Figure 6:**
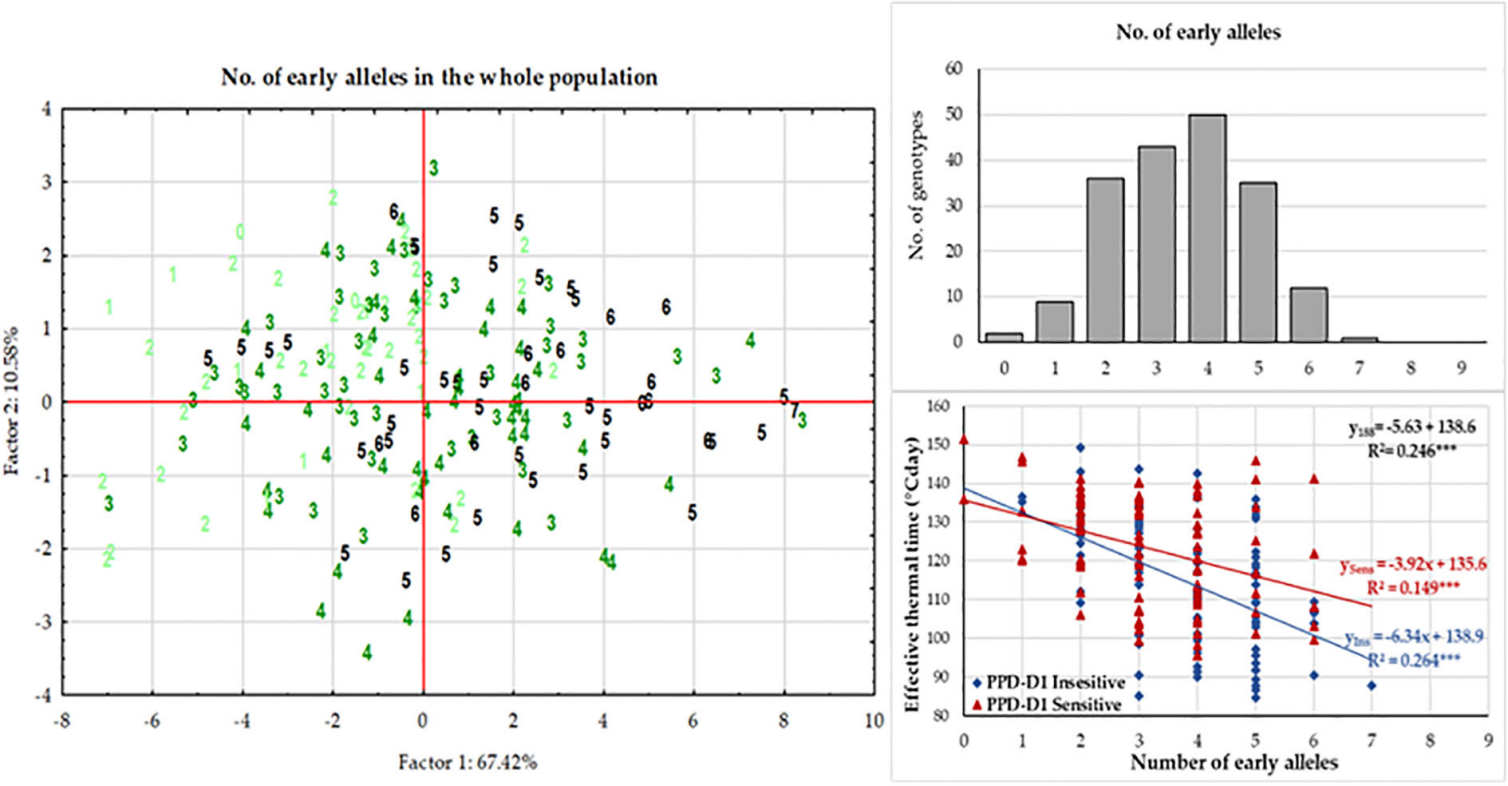
Effects of the number of early alleles in the significant markers on plant development of 188 winter wheat genotypes as represented by the Principal Component analysis on the data matrix of five developmental phases in three seasons and by the association between effective thermal time to first node appearance (Z31).

At each significant marker locus, the allele phase responsible for fastened plant development was identified, and we determined the number of the so-called “early alleles” including *PPD-D1* in the 188 wheat genotypes. The range was between zero and seven, with the majority of the genotypes carrying various combinations of two-to-five early alleles (**Figure 6**). Assuming that all these markers have additive effects, the number of early alleles was used as labels for cases in the plant developmental PCoA (Principal Coordinate Analysis) plot (**Figure 6**). Although no clear pattern was detectable, genotypes with only few early alleles (0-2) tended to be grouped more at the left side of the plot. Regression analyses between the number of early alleles and the ETT values of the developmental phases, however, identified significant moderate associations; R^2^ values at 0.202 (for Z30), 0.246 (for Z31), 0.213 (for Z49), 0.228 (for Z59) and 0.119 (for ZSE), all highly significant. Using Z31 with the highest R^2^ value as an example for illustration, the trend of fastened plant development was observed as the results of more early alleles (**Figure 6**). The number of early alleles in the two allele-phase groups of *PPD-D1* showed overlaps, but the steepness (-6.34 vs. -3.92 ETT/number of early alleles) and the significance (R^2^ = 0.264*** vs 0.149***) of the trend line was much higher in the insensitive genetic background.

### Genetic components of plant development in the group of *PPD-D1* insensitive winter wheat cultivars

**Figure 7:**
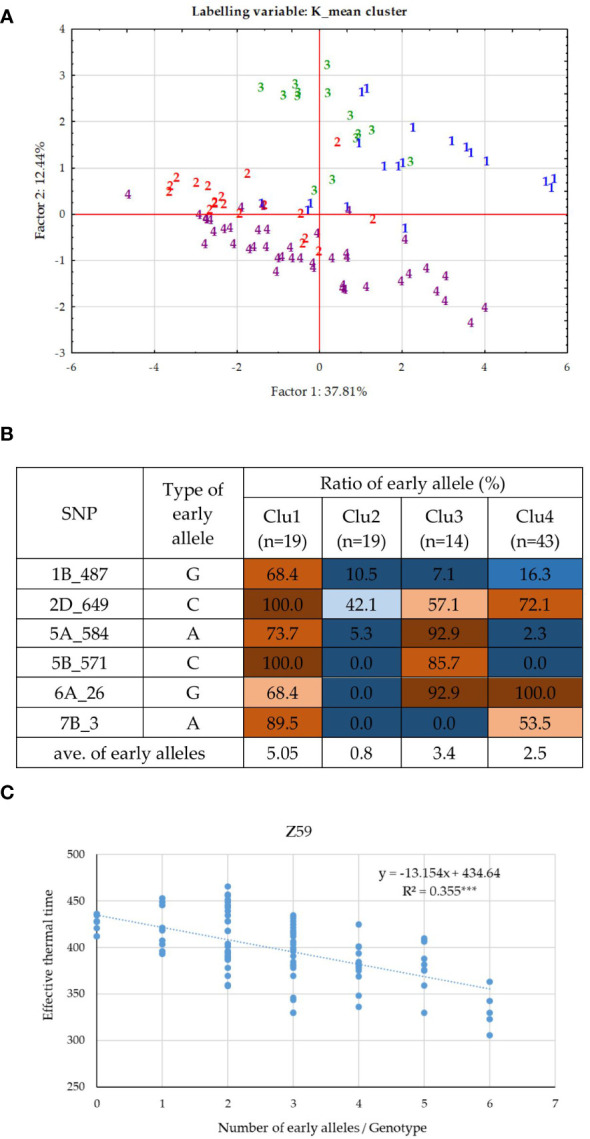
Inner structure of the *PPD-D1* insensitive group of 95 wheat cultivars. **(A)** PCoA on the data matrix of the means of the five consecutive developmental stages and six significant SNPs defining plant development, (labelled by the cluster positions from the K-mean clustering protocol). **(B)** Allele compositions of the four K-mean clusters in the six SNPs defining earliness. **(C)** Associations between the number of early alleles and heading date.

As the effect of *PPD-D1* was prominent and the population for the allele phase of *PPD-D1* was balanced, GWA was also conducted separately on the two subpopulations of the insensitive and the sensitive genotypes. In the case of the insensitive group, 8 significant SNPs of the various developmental phases were identified (**Supplementary Figure 2A**). With the exception of 6A_26M, they were not detectable in the whole population of 188 wheat genotypes. The correlation between these SNPs was observed in the range of 0.00 and 0.37 in absolute value; only one r with a value of 0.76 between 5A_584M and 5B_571M was found. The markers can be again distributed into three significance patterns across the developmental phases (**Figure 4B**). 1A_539M, 4A_9M and 6A_26M influenced the early developmental phases Z30 and Z31, while 1B_487M, 2D_649M and 7B_3M influenced from Z49 to ZSE. The patterns of the remaining two markers 5A_584M and 5B_571M were not similarly accurate, both being detectable at Z30 and again at Z59, to various extents. Applying K-mean clustering on the data matrix of 8 SNPs and 95 insensitive wheat genotypes, the probability of four subgroups was the highest. The appropriateness of the K-mean clustering was checked with Discriminant Canonical Analysis, which proved that 96.8% of the genotypes were placed correctly (F (24,244) = 46.109 p < 0.0000). Two of the eight SNPs, 1A_539M and 4A_9 had no discriminating power, as a result of both becoming rare alleles within the insensitive group (13:82, and 4:91, respectively). To illustrate the inner structures of the insensitive group, both the SNP allele types and developmental traits were included in the matrix for Discriminant analysis (**Figure 7A**). The four clusters were distinctly positioned with a larger distance between two pairs of groups. Thus, Clu1 and 3 were closer to each other and opposite to the other closer positioned Clu2 and Clu4. When the allele compositions of these clusters in the six discriminating SNPs were further analyzed, the uneven distributions of the early alleles became apparent (**Figure 7B**). Clu1 was characterized by the largest presence of early alleles: the average value of early alleles present in the genotypes belonging to this cluster was 5.1 (the maximum being 6). This cluster possessed the highest early allele ratio in four of the 6 SNPs, while Clu3 (closely placed to Clu1 and with an average of 3.4 early alleles) had the highest ratio in the remaining two. The opposite was true for Clu2 (with an average of 0.8 early alleles), which carried no early alleles in three SNPs, and the ratio was around 10% or lower in two other markers. The largest cluster was Clu4 with 43 wheat genotypes, and it was characterized with more mixed allele structure; nevertheless, the lower ratio of early alleles was more prominent overall (with an average of 2.5 early alleles). There was a highly significant positive association between the number of early alleles in a given wheat genotypes (from 0 to 6) and the faster development, as represented with Z59 (**Figure 7C**). As a result, the difference in the developmental speed between the four clusters was also significant at each developmental stage (**Supplementary Table 3**). Clu1 was always the earliest, while Clu2 always the latest, irrespective of the developmental phase. There was, however, a reverse tendency in the case of the two intermediate clusters, Clu3 and Clu4. At the two early phases, Clu3 was as early as Clu1, but as the development advanced, the early development disappeared, and by ZSE, it was as late as Clu2. An opposite pattern was characteristic of Clu4: by ZSE it was as early as Clu1. Among the 95 insensitive wheat cultivars, there were five that carried the early alleles in all the six SNPs, and although four of them were of Chinese origin, they were genetically diverse. ‘Nuo-Maizi’ belongs to cluster 1 in the genetic dendrogram (**Figure 1**), ‘Yumai 21’ and the more closely related ‘Feng You 3’ are in cluster 2, while ‘Yumai 10’ and ‘Briana’ from Romania are in cluster 4. The presence of the late alleles in all the six SNPs was characteristic of 8 cultivars from different geographic locations. In this case, however, a stronger genetic connection was more apparent. Six cultivars belonged to the same genetic cluster (cluster 5), and what is more, four of them were quite closely related (‘Turkmen’ (TR), ‘Jubilejnaja 50’ (UKR), ‘Mv Magma’ (HUN), ‘Pervitsa’ (RUS)), while the other two were less connected within this cluster (‘Mv Toldi’ (HUN) and ‘Mv Amanda’ (HUN)). The other two remaining cultivars were more diverse: ‘Adriana’ (HR) belonged to cluster 2 and ‘Aura’ (RO) to cluster 4.

### Genetic components of plant development in the group of *PPD-D1* sensitive winter wheat cultivars

**Figure 8:**
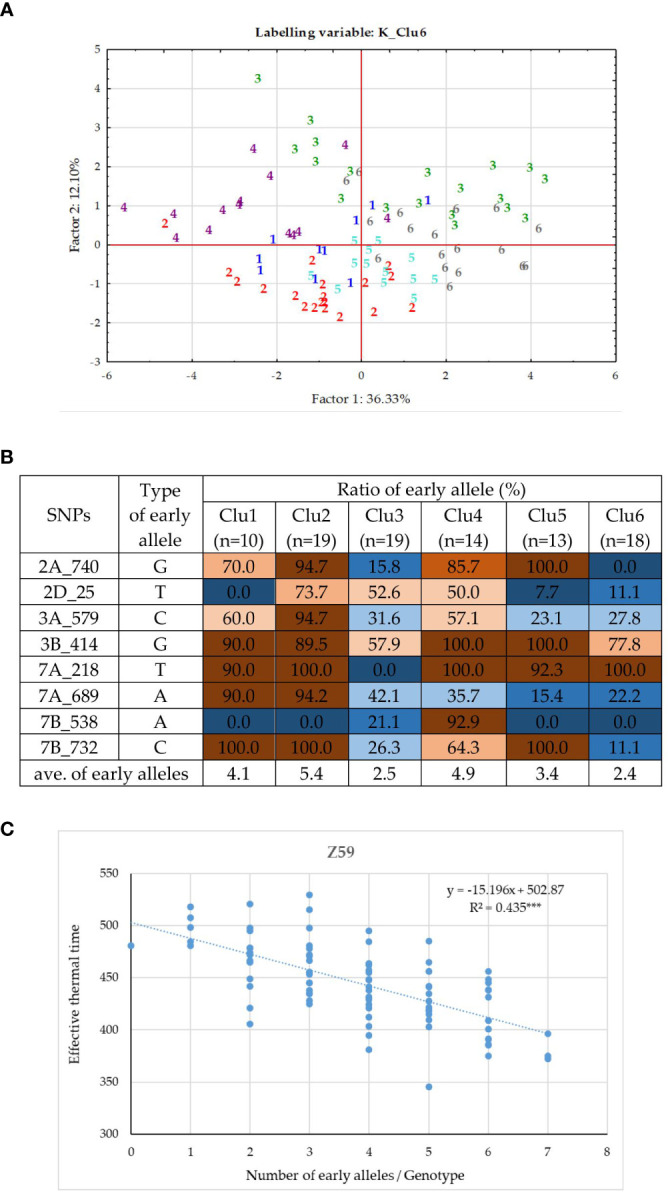
Inner structure of the *PPD-D1* sensitive group of 93 wheat cultivars. **(A)** PCoA on the data matrix of the means of the five consecutive developmental stages and eight significant SNPs defining plant development, (labelled by the cluster positions from the K-mean clustering protocol). **(B)** Allele compositions of the six K-mean clusters in the eight SNPs defining earliness. **(C)** Associations between the number of early alleles and heading date.

Similar analyses were conducted for the *PPD-D1* sensitive group of 93 wheat genotypes. Eight significant SNPs of various developmental phases were identified on six chromosomes (2A, 2D, 3A, 3B, 7A and 7B, **Supplementary Figure 2B**). Of these significant markers, 7B_732 was the same, it was found in the entire population, and 2A_740 was more closely related to 2A_727 identified in the 188 wheat cultivars, but the remaining 6 SNPs were specific to the sensitive group solely (2D_25, 3A_579, 3B_414, 7A_218, 7A_689 and 7B_538). There was no marker overlap between the insensitive and sensitive groups. The correlations between the SNPs were in the range of 0.00 – 0.40. Two SNPs, 2D_25 and 3B_414 were significant determinants of the early developmental phases (Z30 and 31), while four markers, 2A_740, 3A_579, 7A_218, and 7A_689 influenced any of the later developmental phases from Z49 to ZSE (**Figure 4C**). The significance patterns of the remaining two SNPs were unique; 7B_732 was only detectable at Z30, while 7B_538 at ZSE. The 8 SNPs together explained between 16.6% (Z30, significant at P = 0.001 level) and 50.6% (Z59, significant at P=0.0001) of the phenotypic variance. The K-means clustering protocol applied on the data matrix of the 8 SNPs and 93 sensitive genotypes proved the presence of 6 subgroups to be the most appropriate. Discriminant Canonical Analysis proved that 96.8% of the genotypes were again placed correctly (F (40,351) = 27.633 p < 0.0000). In the *PPD-D1* sensitive genotypes, the inner structure was not as apparent as the insensitive ones, which was apparent both in the more complex overlapping patterns of the Discriminant plot (**Figure 8A**) and in the larger mixtures of early and late allele frequencies in the 8 SNPs within each of the six clusters (**Figure 8B**). In general, the members of Clu2 carried the early alleles in the highest frequencies, followed by Clu4, while the presence of the late alleles was the most characteristics to Clu6 followed by Clu3. Nevertheless, the positive associations between the number of early alleles of a given genotype and their earlier developmental patterns were highly significant at each developmental phase (as presented for Z59 in **Figure 8C**). Clu4 was always the earliest at each developmental phase, but the difference from Clu2, the other early group was only significant at the two early phases, Z30 and 31 (**Supplementary Table 4**). Opposite pattern was true to the two late groups; they were similarly late at Z30 and 31, but Clu6 became significantly the latest from Z49 to ZSE. There was only one cultivar, Amor (DEU, from the genotypic cluster 2), with 0 early alleles in the 8 SNPs, and additional 6 cultivars carried only 1 early allele (‘Ellvis’ (DEU, from geno cluster3), ‘NZ4321-114’ (USA, from geno cluster2), ‘Ordeal’ (UK, geno cluster3), ‘Rigi’ (Swish, from geno cluster3), and ‘Balance’ (FRA, geno cluster3)). On the other extreme side, even fewer genotypes were identified with the specific allele combinations, none carried all 8 early alleles, and only three carried 7 early alleles, ‘APS1P-ADE’ (ITA, geno cluster1), ‘ND495’ (USA, geno cluster1), and ‘MVSW33-05’ (HUN, geno cluster1).

### Effects of the genetic components of plant development on morphological and grain- yield related traits

We examined whether the plant developmental SNPs also s impacted any of the morphological and yield- related traits. In order to better facilitate the identification of any possible co-localization, the significance threshold was lowered to –log10(p) = 2.00 instead of 3.00 that was generally used in the GWAS analyses. In the complete 188- wheat- panel, several significant morphological and yield-related MTAs were identified either at or in the close vicinity of the developmental SNPs (**Table 1**). Half of the plant developmental loci influenced several other traits as well. Three of these, including *PPD-D1*, 5B_520 and 2A_27, impacted the morphological traits: plant height (PH), length of last internode (LIN), spike length (EaL), and the number of spikelets (SPIK) in which the role of *PPD-D1* was especially pronounced. The faster developing *PPD-D1* insensitive genotypes tended to be shorter in PH, LIN and in EaL, with fewer numbers of SPIK. The associations were similar for 5B_520 and 2A_27, though to a much smaller extent. However, these developmental and morphological differences were rarely reflected in yield characteristics, and if so, this was highly dependent on the seasonal conditions. A good example for this phenomenon is the effect of *PPD-D1* locus on AET, which reached the significance threshold of 3 in only 1 year, while it was around 2 during the second year, and had no effects during the 3^rd^ year. The 7A_1 locus was the only exception from this pattern, as this locus had no strong influence on morphological traits, with the exception of SPIK, but it contributed significantly to the seed weight of the main spike and to the average thousand kernel weight, and contributed to the average seed weight to a smaller extent. It is important to note, however, that in this locus two completely independent SNPs (r = 0.047^ns^) were responsible for the detected MTAs of the developmental, the morphological, and the yield- related traits.

**Table 1:** Co-localizations of morphological and grain yield related marker-trait associations (MTAs) with the plant developmental loci in the group of 188 winter wheat collections.

Chr.	Plant developmental SNP	co-localizing region (million bp)	Trait^1^	-log10(p)	Weight^2^ (%)	-log10(p) in		
						2013	2014	2015
2A	**2A_27**	27	EaL	2.45	3.5	2.38	1.96	2.70
			LIN	2.85	4.1	**3.22**	2.37	2.70
			PH	2.85	3.8	**3.12**	2.28	2.81
			MSW	2.33	3.5	2.48	1.68	1.52
			ASW	2.24	3.1	2.83	1.23	1.62
			AET	2.42	3.7	2.22	2.46	1.82
			GY	2.38	4.0	0.53	1.82	1.23
2D	***PPD-D1***	20 - 35	**EaL**	**3.08**	4.6	**3.01**	**3.53**	2.77
			**LIN**	**4.03**	6.1	**3.75**	**4.49**	**4.11**
			**PH**	**3.17**	4.3	2.87	2.62	**3.29**
			SG	2.15	2.7	**3.12**	1.71	1.42
			**SPIK**	**5.35**	8.6	**4.53**	**7.21**	2.42
			AET	2.03	3.0	2.07	**3.25**	0.29
4A	**4A_570**	577 – 617	**EaL**	**3.04**	4.6	**3.03**	**3.76**	2.79
5B	**5B_315**	315	AET	2.79	4.4	2.07	**3.29**	1.89
5B	**5B_520**	515 – 547	**LIN**	**3.65**	6.1	2.91	**3.67**	**3.45**
			PH	2.85	3.8	2.98	**3.13**	2.80
			SG	2.01	2.5	2.24	2.61	1.48
			SPIK	2.72	3.8	1.89	**3.02**	2.28
6A	**6A_26**	24 – 51	**SG**	**3.20**	4.5	1.77	**3.11**	**3.68**
7A	**7A_1**	0.5 – 1.9	SPIK	2.40	3.3	2.87	1.42	2.11
			MS	2.29	3.5	2.29	1.33	1.85
			**MSW**	**3.76**	6.3	**3.44**	2.18	**3.18**
			AS	2.02	2.6	1.18	1.48	2.11
			ASW	2.88	4.3	2.01	1.39	2.88
			**AET**	**3.20**	5.2	2.36	**3.80**	**3.53**
			GY	2.02	3.3	1.07	0.89	1.69
7B	**7B_732**	648 – 732	SPIK	2.95	4.2	2.33	2.38	**3.09**

^1^ Trait abbreviations: EaL, length of main ear; LIN, length of the last internode; PH, plant height; MSW, seed weight in the main ear; ASW, average seed weight; AET, average thousand kernel weight; GY, grain yield/ plant; SG, speed of intensive stem elongation; SPIK, spikelet number in the main ear; AS, average seed number per spike.

^2^ expressed in % of variance explained and calculated as R^2^ of model with SNP – R^2^ of model without SNP. Bold numbers indicate LOD values greater than 3.

In addition to the former groups of loci, there were four developmental loci, which showed associations with only one other trait (**Table 1**). The largest additional effect was characteristic to 6A_26, which significantly influenced SG, the speed of intensive stem elongation. 4A_570 contributed to the spike length, while 7B_732 contributed to the numbers of spikelets. These three associations were independent of the seasonal conditions. Finally, also, 5B_315 contributed to AET, but this association depended more on the seasonal conditions.

In the next step, we also examined whether the clusters with various numbers of earliness alleles show any differences in the morphological and yield-related traits within the insensitive or sensitive *PPD-D1* genetic backgrounds. The developmental differences of the four clusters of *PPD-D1* insensitive wheat genotypes had only minor effects on the morphological traits and no significant effects at all on the yield-related traits (**Supplementary Table 3**). Similar was the case for the six *PPD-D1* sensitive clusters. Although they represented distinctly different developmental patterns, again, with a few exceptions, there were no significant differences between these developmental clusters either in morphological or in yield-related traits (**Supplementary Table 4**).

### Plant developmental and yield-related traits of the groups combining extreme numbers of early versus late alleles in the genetic background of the two *PPD-D1* allele phases

**Figure 9:**
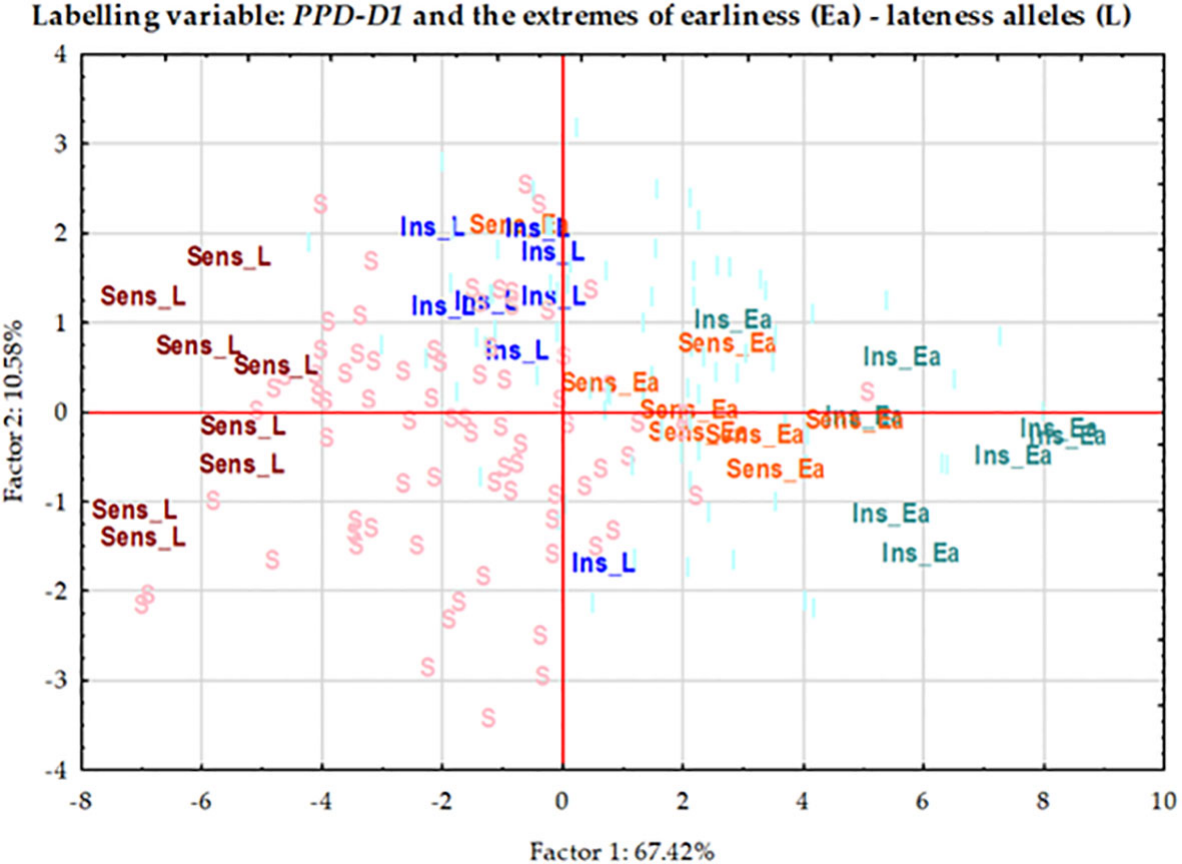
Principal Component analysis carried out on the phenotypic data matrix of the effective thermal times of five consecutive developmental phases across three seasons in the group of 188 winter wheat genotypes, where 32 wheat genotypes are highlighted representing the four combinations of *PPD-D1* allele phase and the extremity of early vs. late developmental alleles.

In order to further examine the possible effects of plant developmental patterns on yield-related traits, we selected a subset of genotypes that were balanced between the *PPD-D1* allele phase and the two extremities in the number of early alleles. The genotypes in the early groups equaled or were close to maximum of the possible early alleles and those in the late group equaled or were close to 0 (**Supplementary Table 5**). Thus, we created four groups; *Ins early*, *Ins late*, versus *Sens early* and S*ens late*. As the highest number of extreme genotypes in the *Ins late* allele group was eight, the remaining three groups were also equalized to eight in order to create the balanced set. The selection was based on the next closest number of early or late alleles and on the Z59 values (**Supplementary Table 5**). Including the information on these 32 genotypes into the PCoA graph of *PPD-D1* allele type, it revealed the genetic bases of the overlapping in the plant developmental patterns of the *PPD-D1* insensitive and sensitive wheat genotypes (**Figure 9**). Two additional major characteristics are noted. First, that the plant developmental patterns of the early vs. late groups may be clearly recognized, irrespective of the allele phase in *PPD-D1*, but the difference between the two different groups was larger in the *PPD-D1* sensitive background. In addition, the presence of high numbers of early alleles in the *PPD-D1* sensitive background resulted in significantly faster plant development compared to those of insensitive *PPD-D1* lines lacking any of the early alleles. In fact, in this selected subset of 32 wheat cultivars, the effects of early alleles were more significant determinants of plant development than the *PPD-D1* allele phase, especially from the appearance of the first node to heading (**Table 2**). The four groups significantly differed from each other in all the five developmental phases in the following order from earliest to latest: *Ins early*, *Sens early*, *Ins late*, and *Sens late*. The four groups showed also highly significant differences in the spikelet number and in the average thousand kernel weight (**Table 2**). The *PPD-D1* allele phase and the number of early alleles similarly influenced the number of spikelets, which increased both due to the sensitive and to the late alleles. AET, however, was mostly influenced by the *PPD-D1* allele. It was the highest in the two insensitive groups, irrespective of the number of early vs. late alleles, and it significantly decreased in the sensitive background, especially when it was accompanied by late alleles.

**Table 2:** Descriptive statistics of plant developmental, morphological and yield-related traits and the group averages in 32 winter wheat genotypes representing the early and late groups within each allele phase of *PPD-D1*.

Trait	Variance components (SS%)^1^				Ins_ea n = 8	Ins_late n = 8	Sens_Ea n = 8	Sens_late n = 8	P level of the model
	PPD-D1	Ea alleles	PPD x Ea	Within group					
Z30	20.3***	49.1***	0.0	30.7	107.2a^2^	140.2bc	128.4b	161.7d	2.4E-07
Z31	11.6***	65.4***	0.3	22.7	92.9a	128.4c	109.2b	139.9d	3.6E-09
Z49	24.8***	67.7***	0.5	7.1	273.7a	356.4c	320.7b	418.8d	3.2E-16
Z59	24.2***	68.2***	1.5*	6.1	341.2a	425.1c	385.2b	498.5d	4.2E-17
ZSE	29.2***	53.1***	2.6*	15.1	407.4a	472.9c	451.2b	553.6d	1.3E-11
Z3031	12.2+	0.0	1.4	86.4	14.3	11.9	19.2	21.8	0.243
Z4930	21.4***	62.9***	1.1	14.6	166.4a	216.2c	192.3b	257.1d	8.4E-12
Z4931	29.6***	58.6***	1.8*	10.0	180.7a	228.1c	211.4b	278.9d	4.0E-14
Z5949	3.9	16.3*	11.9*	67.8	67.5a	68.7a	64.5a	79.7b	0.011
ZSE59	0.7	12.0*	0.8	86.5	66.2b	47.8a	66.0b	55.1ab	0.248
ZSE30	32.1***	49.5***	6.4*	38.0	300.0a	332.7b	322.8b	391.9c	1.9E-07
PH	0.5	7.6	5.5	86.5	66.3	75.5	71.8	72.6	0.246
LIN	0.8	4.3	10.5	84.5	27.1	31.7	29.2	28.1	0.187
SPIK	21.3***	34.5***	0.0	44.1	19.4a	21.6b	21.1b	23.4c	3.5E-05
SSP	6.7	4.9	7.8	80.5	2.76	2.42	2.40	2.44	0.103
MS	0.4	4.0	7.8	87.8	53.4	52.3	50.5	56.9	0.295
MSW	17.3*	0.1	2.9	79.8	2.66	2.49	2.16	2.28	0.092
AS	2.6	13.7*	7.7	76.0	38.2ab	39.3ab	36.9a	44.5b	0.050
ASW	14.4*	1.4	0.6	83.7	1.77	1.79	1.50	1.61	0.167
AET	47.1***	5.5	2.2	45.1	46.6c	45.6c	40.6b	36.3a	4.8E-05
GY	9.8+	0.3	0.1	89.8	14.7	14.9	12.6	13.1	0.380

^1^ significant at *, **, *** P = 0.05, 0.01, and 0.001 levels, respectively.

^2^ the average values of the four groups followed by the same letters within one row are not significantly different from each other at P = 0.05 level.

**Figure 10:**
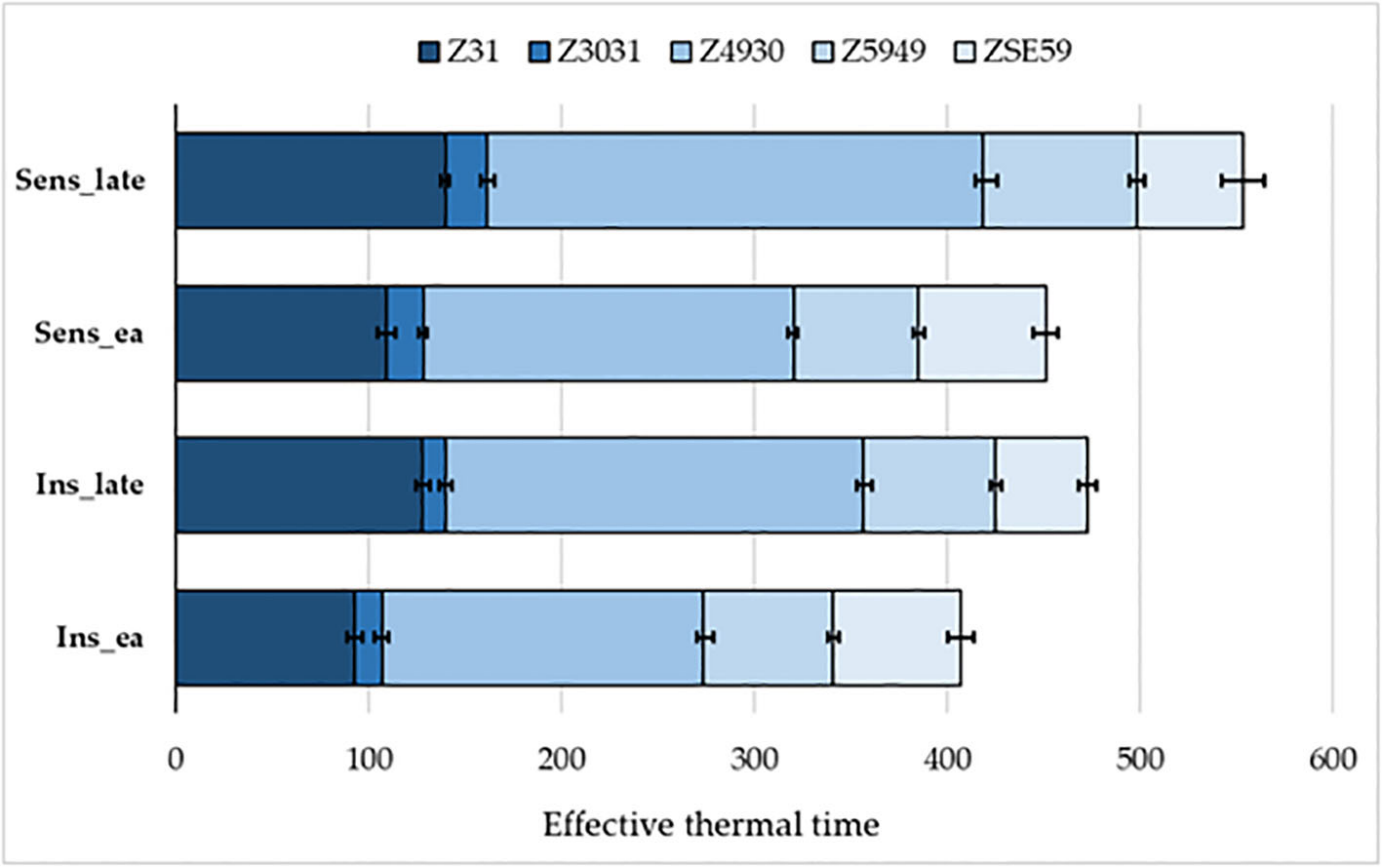
Developmental patterns of the four extreme groups combining the *PPD-D1* allele phase (Insensitive – Sensitive) together with the allele phases in the most significant SNPs of plant development (highest (early) or lowest (late) number of early alleles). Error bars are standard deviations within each group.

The acceleration effects of the early alleles on plant development were not proportional across the consecutive developmental phases (**Figure 10**). The variance analyses of the various developmental intervals (**Table 2**) revealed that the allele phase together with the extremes of early alleles had the strongest influence on the intervals between first node appearance and booting (Z4931) and between the start of the intensive stem elongation and booting (Z4930), followed by the length of intensive stem elongation (ZSE30). In all these cases, the influence of early alleles was stronger than that of *PPD-D1*, especially for the first two intervals. In the early groups, these intervals were significantly shorter than in the late groups, irrespective of the allele phase in *PPD-D1*, though the difference between early and late were always greater in the sensitive background. For the remaining intervals, the within-group variance was the major factor and only a minor influence of earliness was detected for the later developmental intervals including the length between booting and heading (Z5949) and between the heading and the end of intensive stem elongation (ZSE59). In the case of Z5949, the difference between the early and late groups was only significant in the *PPD-D1* sensitive background, while the opposite was true for ZSE59.

**Figure 11:**
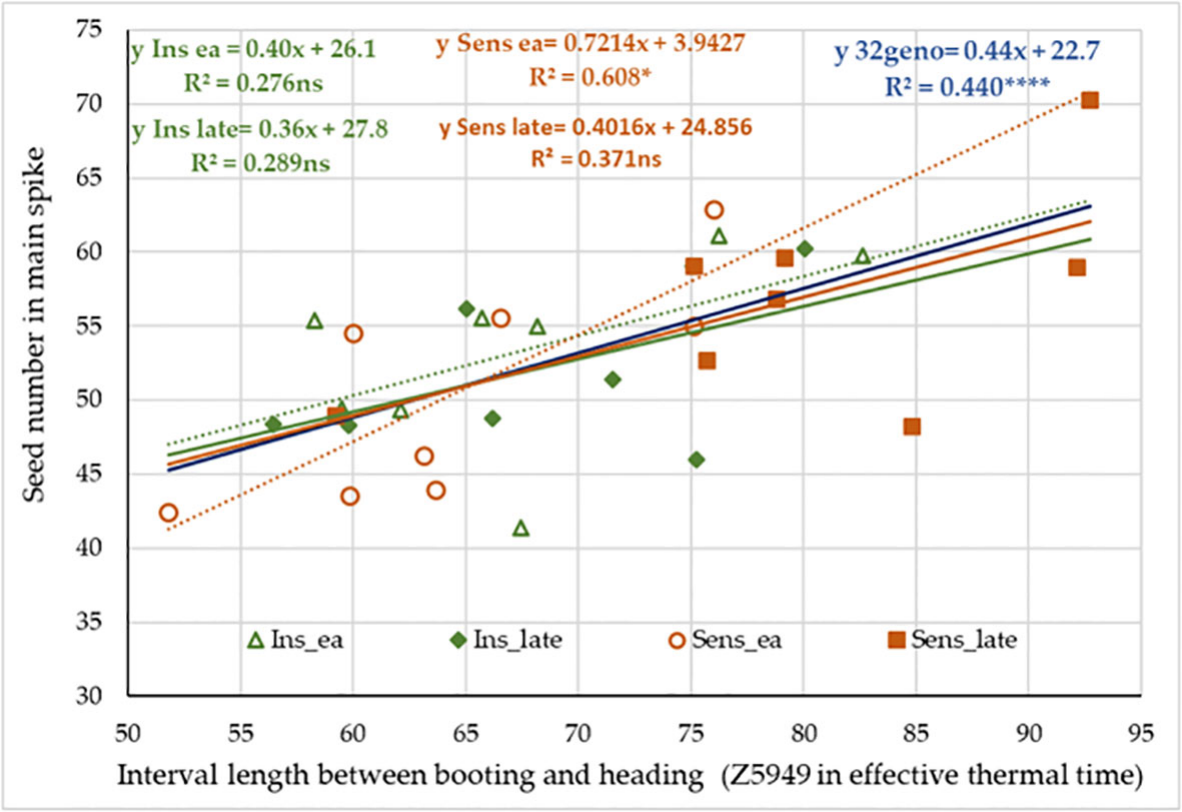
Effect of the interval length between booting (Z49) and heading (Z59) on the number of seeds in main spike in the context of *PPD-D1* allele phase and the possible maximum number of early – late alleles in the significant developmental SNPs.

On the data matrix of the 32 selected wheat genotypes, we also examined the possible associations between the lengths of developmental intervals and various traits (**Table 3**). The interval between Z30 or Z31 and Z49 showed a strong positive correlation with the spikelet number, a medium negative correlation with the average thousand kernel weight, and a weak positive correlation with the average seed number. These data show strong correlation with the results of the variance analysis conducted on the four groups. More interesting is the case of Z5949, when the variance analysis indicated only an insignificant effect of early alleles when comparing the variance between and within the four groups. While running regressions in the group of 32 genotypes however, the length of this interval proved a significant determinant of seed numbers both in the main and side ears, and in parallel, it significantly contributed to seed weight, but not to the thousand kernel weight. All these associations were positive, meaning that a longer interval between booting and heading resulted in more seeds/ear, and as a result, more seed weight. When the 32 genotypes were analyzed separately by the four groups, the trends of three groups were similar to the main trend (**Figure 11**). *Sens early* was the only group with a steeper association; here a unit increase in the interval length between Z49 and Z59 led to 80% more seeds on average, than in the other three groups.

**Table 3:** Associations between the lengths of developmental intervals and the morphological and yield related traits in 32 winter wheat genotypes representing the early and late groups within each allele phase of *PPD-D1* (R^2^ - variance explained and P – its significance at +, *, **, *** P = 0.1, 0.05, 0.01, and 0.001 levels, respectively).

Trait	Z3031		Z4930		Z4931		Z5949		ZSE59		ZSE30	
	R^2^	P	R^2^	P	R^2^	P	R^2^	P	R^2^	P	R^2^	P
PH	0.091	+	0.096	+	ns		ns		ns		0.119	*
LIN	ns		ns		ns		ns		0.134	*	ns	
SPIK	ns		0.597	****	0.590	****	0.195	*	ns		0.456	****
SSP	ns		0.147	*	0.101	+	0.107	+	ns		ns	
MS	ns		ns		ns		0.440	****	ns		ns	
MSW	ns		ns		ns		0.216	**	ns		ns	
AS	0.091	+	0.154	*	0.215	**	0.386	***	ns		0.160	*
ASW	ns		ns		ns		0.199	*	ns		ns	
AET	0.170	*	0.206	**	0.306	**	ns		ns		0.228	**
GY	0.129	*	ns		ns		0.140	*	ns		ns	

Trait abbreviations: PH, plant height; LIN, length of last internode; SPIK, spikelet number per main ear; SSP, seed number per spikelet in main ear; MS, seed number in main ear; MSW, seed weight in main ear; AS, average seed number; ASW, average seed weight; AET, average thousand kernel weight; GY, grain yield per plant. ns, not significant.

## Discussion

With this study, our aim was to evaluate the contribution of the genetic components determining the dynamics of plant development to grain yield and its related traits under field conditions in the course of a multi-seasonal experiment. For this purpose, a GWA population of 188 winter and facultative wheat genotypes of diverse geographic origin was described. This experiment was novel because the phenotypic characterization included the plant developmental dynamics evaluation across five consecutive developmental phases: from first node appearance to intensive stem elongation, and it was compared with the yield- related trait measurements. The GWA panel was genotyped with not only different sets of SNP platforms, but it was also screened for the allele compositions in several major plant developmental genes *via* the application of gene specific primers and/or KASP-based copy number predictions. These markers were in most cases functional ones. Thus, genes of photoperiod sensitivity (*PPD-B1*, *PPD-D1*), vernalization response (*VRN-A1*, *VRN-B1*, and *VRN-D1*), and earliness per se (*TaFT3-1A*, *TaFT3-1D* and *TaELF3-1D*), dwarfing genes (*Rht-B1* and *Rht-D1*) and the presence-absence of the 1B/1R translocation were built into the LD (Linkage Disequilibrium) map used for GWA. From these genes, however, only the significant effect of *PPD-D1* was directly detected in the entire population of the 188 wheat genotypes in all the seasons. In addition, the significant but minor effect of an SNP marker closely linked to *VRN-A3* was also identified in the complete panel. In the flowering time literature, the effects of known plant developmental genes with smaller effects often stay undetected under field conditions (Basile et al., 2019). This phenomenon may be the result of the complexity of various environmental factors prevailing at a given location and of the environmental saturation of these genes (Trevaskis et al., 2007; Cockram et al., 2007). In addition, it may be the result of possible complex epistatic interactions between the various gene alleles, which may prevent the identification of the actions of minor flowering genes. The latter possibility is also well demonstrated by the identified significant sets of MTAs unique to the two allele types of *PPD-D1*.

### *PPD-D1* is the major driving force of plant development from early spring in winter wheats

For *PPD-D1*, the functional marker used for mapping depicts the large deletion in the promoter region that renders the genotypes insensitive to photoperiod, resulting in fastened plant development both under short and long photoperiods (González et al., 2005a; Beales et al., 2007; Bentley et al., 2013). Analyzing the population structure × geographic origin × allele phase in the plant developmental genes, we found that both the sensitive and insensitive alleles of *PPD-D1* were present in the cultivars of the different megageographic regions, but their ratio varied. The majority of West-European and American cultivars carried the sensitivity allele (77.1% and 63.6%, respectively), while the majority of East-European, South-European, and Asian cultivars carried the insensitivity allele (88.9%, 87.5%, and 62.5%, respectively). For the Central-European wheats, the ratio of insensitive and sensitive alleles was similar (52.1% and 47.9%, respectively). In spite of these definite differences between wheats from different geographic locations, the distribution of the two alleles of *PPD-D1* was fairly even across the various subclusters of the complete wheat collection due to the specific characteristics of the population structure.

In accordance with the already-published results, we identified this gene as the most significant component of plant development in our field experiments conducted over several seasons (Worland et al., 1998; González et al., 2005b; Langer et al., 2014; Benaouda et al., 2022). The effect of *PPD-D1* was significant during the plant development from early spring, but its weight became most pronounced with the later developmental phases, similar to *PPD-H1* (Karsai et al., 1997; González et al., 2005a; Prieto et al., 2018). In spite of its basic determining role, *PPD-D1* explained only a modest portion of the phenotypic variation this wheat collection presented (between 12.1% and 18.9%), and large differences remained within both the insensitive and sensitive subgroups. This result somewhat contradicts the results of a large set of mostly European winter wheat GWA panel, where *PPD-D1* was the sole largest significant determinant of the heading date (Langer et al., 2014). The majority of that population carried the sensitivity allele that strongly coincided with the geographic location as opposed to the *PPD-D1*-balanced population from more diverse geographic locations, with possibly larger allelic variations in the minor flowering time loci in the present study. Due to the limited genotypic diversity, the greater impact of *PPD-D1* was also detected in biparental populations or near-isogenic lines segregating for *PPD1* (Karsai et al., 1997; González et al., 2005a; Borrás-Gelonch et al., 2012; Prieto et al., 2018),. Kiss et al. (2014) examined the direct effects of the major plant developmental genes on plant development under field conditions in more than 600 winter wheats, not taking into account the population structure. In their scenario, *PPD-D1* alone was found to explain 25%–28% of the phenotypic variation and the gene model prediction including the main allele types of the three *VRN1* and the two *PPD1* genes increased this ratio to a maximum of 37.9%, similar to that detected by Eagles et al. (2010) in a large set of Australian wheats.

The lower phenotypic ratio of *PPD-D1* found in this experiment, however, may also be the result of the use of effective thermal time instead of chronological time, which compensates for the temperature differences across seasons. Benaouda et al. (2022) demonstrated that use of Julian days (days elapsed from 1st January) versus spring growing degree days resulted in significant differences in the data analyses of the heading date between the general effects of six different locations of latitudinal cline in Germany. When the two approaches were compared, they found that the latitudinal response to day length showed an opposite trend to that of Tmax; thus, they concluded that the seasonal variation in temperature at low latitudes may replace the effect of seasonal variation in the photoperiod. Their GWA population, however, consisted of mostly German cultivars carrying the sensitivity *PPD-D1* allele. It had already been proven that *PPD-D1* determines plant development *via* the sensing photoperiod and the ambient temperature, and wheat genotypes with the sensitivity allele more frequently responded with delayed plant development due to higher ambient temperature (Kiss et al., 2017; Basavaraddi et al., 2021). Hungary is located at latitudes lower than the German location of the lowest latitude in their study but high enough to experience a seasonal photoperiod fluctuation of cca. 8 h between the winter and summer solstices. Thus, *PPD-D1* was a significant component of development with varying intensities during all three seasons.

As our GWA population was balanced for the allele phase of *PPD-D1*, the association analyses was conducted not only in the entire population but also separately in the two *PPD-D1* allele- phase subgroups. This approach identified not only significant additional plant developmental loci that were independent in their actions from *PPD-D1* (they were detected in the entire population but not in any of the subgroups) but also loci detectable only in one of the two *PPD-D1* subgroups with no overlaps between them. Thus, our results demonstrate that *PPD-D1* not only directly but also indirectly determines plant development through epistatic interactions that influence minor developmental loci. The *PPD-D1* allele phase was decisive on the sets of loci with significant effects on plant development (Borrás-Gelonch et al., 2012; Guo et al., 2018b; Basavaraddi et al., 2021; Benaouda et al., 2022).

### Minor loci of plant development

In addition to *PPD-D1*, the significant effects of eight minor loci were also detected in the entire population of 188 wheat genotypes, and eight–eight loci in the two subpopulations of the two allele phases of *PPD-D1*. No overlap of the minor loci between the two subgroups of *PPD-D1* was detected, and only one and two loci were the same between the complete collection and the insensitive subgroup or between the complete collection and the sensitive subgroup, respectively. We identified altogether 21 minor loci of plant development, each explaining individually only a small portion of the phenotypic variance (between 3.0% and 6.1%). The candidate genes responsible for these effects are not yet determined in most cases. In the *PPD-D1*-insensitive subgroup, however, significant SNP markers were detected in the close vicinities of *VRN-A1* (5A), *VRN-B1* (5B), and *VRN-B3* (7B), which were not apparent either in the complete set of 188 wheat genotypes or in the *PPD-D1*-sensitive subgroup (**Supplementary Table 6**). This may indicate the epistatic interactions between the specific allele phases of *VRN1*, *VNR3*, and *PPD1* (Karsai et al., 2008; Kiss et al., 2014; Alipour and Abdi, 2021; Cao et al., 2021).

In most cases, the allele type conferring earliness may be clearly distinguished, and it was proven that the earlier alleles were present in a wheat genotype, the earlier its heading within the complete collection or within each subgroup (Sheoran et al., 2019; Jung et al., 2021). Thus, it is not surprising that the combined phenotypic effects of these loci could have amounted to as high as 50.6% of the variance explained. Only four of the minor loci were detectable throughout the plant development, while the early developmental (Z30, Z31) or later developmental phases (Z49, Z59, and ZSE) were each significantly influenced by eight–eight different loci. Limited data are available on the genetic determinants of the different developmental phases and the various interval lengths under field conditions. Together with our findings, they confirm that the genetic regulation of the various developmental phases is controlled by partially different sets of genes (Borrás-Gelonch et al., 2012; Gonzalez-Navarro et al., 2016; Dreisigacker et al., 2021). In this GWA population of 188 genotypes, large variations were detected between the genotypes in the combination of the early–late alleles at these 21 minor loci, which prevented the exact identifications of the possible interactions between them and between the major plant developmental gene alleles. Thus, the dissection and further analyses of these developmental phase–specific minor loci can significantly contribute to fine-tuning and growing location–specific engineering of plant development.

Several factors render this study difficult to correlate to previously published data (Fernández-Calleja et al., 2022; Yang et al., 2021). These factors include the type of SNP platforms used for genotyping, the genetic backgrounds and the specific haploblock structures of the individual GWA populations, and the large differences in the networks of environmental factors specific to the individual experimental locations and seasons. It is even more difficult to correlate with the results of previous studies that were based on the linkage map distances in cm (Yang et al., 2021). Pang et al. (2020) using genotype-by-sequencing for a large wheat association panel from China of more than 700 genotypes established the LD decay (r^2^ ≤ 0.2) being cca. 50 million bp for the whole genome with a range between 38 (A and B genomes) and 74 Mb (D genome). For assuming a developmental locus being the same, we considered the actual physical position of published significant SNPs along with the actual physical length of the given chromosome, and for MTAs within 5% of the total chromosome length, we considered to depict the same locus (Ward et al., 2019; Pang et al., 2020). The possible overlaps between our results, and the previously published results are demonstrated in **Supplementary Table 5**. In general, most of the loci identified in this study have already been published with various frequencies as being significant genetic components of the heading date and/or maturity time (for references, see **Supplementary Table 6**). This was also true for the loci that we identified only in the early plant developmental phases. The only exception was 7A_218 that contributed to Z49 in the *PPD-D1* sensitive subgroup under conditions in Hungary. However, no reference was found with the same sets of minor loci. Generally only one or a very few loci overlapped between any two studies. These similarities were mostly independent of growth habits, the geographic origins of the genotypes, the megaenvironments of the experimental locations, or possible prevailing abiotic stresses (for references, see **Supplementary Table 6**). The application of GWAS in elite germplasms is generally limited to the identification of smaller-effect MTAs, as major-effect MTAs might have already become fixed within the modern wheat cultivars (Salvi and Tuberosa, 2015). Thus, in modern wheat GWA populations originating from the same megaenvironment, some alleles of the major plant developmental genes may be fixed due to the breeding activities for better adaptation (Eagles et al., 2010; Kiss et al., 2014; Alipour and Abdi, 2021; Benaouda et al., 2022). However, our results also demonstrate that the allele phases of the major plant developmental genes determine the sets of minor developmental loci effective on plant development and are thus detectable. This limitation can be overcome by using a very diverse association panel composed of landraces and/or modern wheat cultivars from different geographic locations (Cseh et al., 2021). Our GWA population is geographically diverse as reflected by the allele diversity in the major plant developmental genes. This diversity helped to identify this set of minor plant developmental loci.

In addition, the rather-random nature of coincidences between the developmental loci identified under diverse circumstances may highlight the intricate and complex regulating systems of plant development where strong interactions also exist between various environmental stimuli and various elements of the gene cascades of developmental regulation. The environmental conditions of our field-sown experiments were close to optimal for wheat development, and only the systematic phenology and the exclusion of *PPD-D1* detected the effects of several minor loci. Here, we have shown that the various combinations of *PPD-D1* allele phases with the early or late alleles of minor plant developmental genes may lead to significantly altered interval lengths between any of two consecutive developmental phases, which confirm previous findings (Gonzalez-Navarro et al., 2016; Dreisigacker et al., 2021). Thus, unfavorable environmental conditions at any stage of plant development may strengthen the effects of the corresponding minor loci leading to their individual location–specific detection even in the heading date and maturity, irrespective to the megaenvironments.

### Effects of *PPD-D1* and the minor plant developmental loci on morphological and yield-related traits

It is well documented that the *PPD1* photoperiod sensitivity genes in cereals influence not only the rhythm of plant development but also several morphological traits including plant height and some aspects of spike morphology (Boden et al., 2015; Guo et al., 2018b; Prieto et al., 2018; Achilli et al., 2022). In general, allele phases fastening plant development result in shorter plant stature and more lax ears with fewer spikelets. In the wheat *PPD-D1* gene, it is the insensitivity allele that enhances plant development both under short and long photoperiods, which results in these pleiotropic effects (Prieto et al., 2018). *PPD1* may influence several yield-related traits including the seed number per ear and thousand kernel weight and, as a final result, grain yield itself, but the experimental data are more controversial in this aspect (Liu et al., 2020; Gol et al., 2021; Jung et al., 2021; Achilli et al., 2022; Fernández-Calleja et al., 2022; Ding et al., 2022). The magnitude of the differences in yield-related traits between the insensitive and sensitive allele type groups may depend strongly on the type of the genetic populations (bi-, multiparental, or association panels) and/or on the environmental conditions of the experimental setup (controlled or field-grown experiments with seasonal and location effects in the case of the latter). Worland et al. (1998) stated that photoperiod insensitivity may benefit grain yield in years and locations with hotter and drier summers. Several experiments revealed the importance of the *PPD-D1* insensitivity allele in abiotic stress tolerance, mostly *via* avoiding the stressful periods through accelerated plant development, which led to earlier heading and ripening with a decreased seed number per spike and, in some cases, higher thousand kernel weight (Liu et al., 2020; Alipour and Abdi, 2021; Dreisigacker et al., 2021). In this process, not only is the photoperiod an important environmental indicator but also the ambient temperature during the phase of intensive stem elongation (González et al., 2005b; Borrás-Gelonch et al., 2012; Kiss et al., 2017). The start and speed of intensive stem elongation accelerate with a higher rate in insensitive genotypes as the ambient temperature level arises. Under favorable conditions, however, those same attributes can be disadvantageous for yield formation, which explains the strong environmental dependence of *PPD1* on grain yield (Liu et al., 2020). Thus, the indirect effects of *PPD1* on yield-related traits may appear mostly as causal consequences of altered plant developmental patterns under unfavorable growing conditions that occur at any time from the start of the intensive stem elongation for any of the two *PPD1* allele phases (Fernández-Calleja et al., 2022).

In complete agreement with data found in the literature, in this set of experiments, the effect of *PPD-D1* was clearly detected on several morphological parameters including plant height, last internode length, ear length, and the number of spikelets per ear but not on yield-related traits. For minor developmental loci, however, no or only weak additional trait associations were detected in the same region, with the exception of three loci. In their regions, one additional morphological parameter was located in addition to the developmental trait associations, which were the length of the last internode (5B_520), ear length (4A_570), and the speed of intensive stem elongation (6A_26). Neither comparison of the yield-related parameters of the developmental pattern–based clusters nor those of the combination of *PPD-D1* allele phases with earliness resulted in any stronger detections of possible associations between the plant developmental loci and yield parameters. The possible explanation is that the 3-year experiment did not include extremes for the prevailing climatic factors and any adverse environmental effects possibly occurred only for shorter periods and/or were not sufficiently powerful to alter the plant developmental patterns, which may have led to significant changes in the yield components.

### Combinations of earliness and photoperiod sensitivity

As large variances were found within each *PPD-D1* allele class in plant development, which was due to the actions of several minor developmental loci, we further examined how combining the extremes of earliness–lateness with photoperiodic reactions may affect plant development. Our results highlighted that the large within-group variances of the two *PPD-D1* classes was mostly the result of the actions of these minor loci. Actually, the combined roles of the minor loci affected the length of the developmental interval between the start of the intensive stem elongation and heading more than *PPD-D1* itself. The length of this interval impacted several yield-related traits, including the spikelet number, seed number per spikelet, average seed number per spike, and average thousand kernel weight. These results confirm the theory that manipulating the developmental interval lengths *via* a conscious combination of various genetic components of plant development may lead to increased yield with a similar time frame of the heading date (Borrás-Gelonch et al., 2012).

Combining the opposite characters of *PPD-D1* and the minor loci into one genetic background seems especially advantageous in light of the climate change as it can ensure a more flexible adaptation strategy throughout the growing period. Building early alleles in a *PPD-D1*-sensitive background, for example, may contribute to better winter survival and summer abiotic stress avoidance. Due to photoperiod sensitivity, the vegetative period is longer, which, in addition to larger biomass, represents a safeguard against late-spring cold spells as the early-spring photoperiod sensitivity prevents precocious stem elongation, a scenario that may occur in higher frequency due to the warmer winters. The longer vegetative period can then be effectively counterbalanced with the early alleles of minor loci that shorten the stem elongation phase, thus helping the plants avoid the drought of early summer, which may positively affect thousand-kernel weight. The opposite combination of *PPD-D1* insensitivity with the late alleles of minor loci may benefit from the generally fast development combined with a longer intensive stem-elongation phase. Central-Europe exemplifies the effectiveness of this scheme as the year-to-year climatic fluctuations are characteristic of this region due to its location in the intersection of three climatic zones (Continental, Mediterranean, and Oceanic). Thus, the cultivars bred and successfully grown in this area are quite diverse for the *PPD-D1* allele phase and their combination of late and early alleles of various minor developmental loci. All these are due to selections for more locally adaptable forms, with stable yielding abilities without being aware of the genetics behind. To utilize the manipulation of developmental processes fully, it is a prerequisite to dissect the exact natures of these loci within the regulating gene cascade of plant development.

## Conclusions

In a multiseasonal field experiment of a winter wheat GWA panel balanced for the allele phase of the *PPD-D1* photoperiod sensitivity gene, we proved that although *PPD-D1* is the single strongest genetic component of plant development, the influence of the sets of minor alleles is significant and contribute to a larger-scale level. Some minor loci are independent of the *PPD-D1* allele phase, while others function in possible epistatic interactions with *PPD-D1* and become detectable only at the specific *PPD-D1* allele phase. The contrasting combination of the early-late alleles of the minor loci with *PPD-D1* insensitivity–sensitivity alleles may lead to a series of developmental range that may be utilized to ensure a greater ecological plasticity of plant development. *PPD-D1* together with the minor loci also contribute to several morphological traits, but their effects on yields and yield-related traits depend more on the environment. The indirect effects of *PPD-D1* on yield-related traits may appear mostly as causal consequences of altered plant developmental patterns under unfavorable growing conditions that may occur randomly from the start of the intensive stem elongation for any of the two *PPD-D1* allele phases.

## Data availability statement

The original contributions presented in the study are included in the article/**Supplementary Material**. The genotypic data presented in this study were generated using a commercially available Illumina Infinium 15k wheat SNP array whose details can be found at https://www.traitgenetics.com/index.php/service-products. The complete data set of 7273 polymorphic markers used to perform the GWAS analyses is also provided in the **Supplementary Material**. Further inquiries can be directed to the corresponding authors.

## Author contributions

Conceptualization, TK and IK; methodology, TK, IK, and AC; validation, TK, IK, and AC; investigation, TK, AH, KB, ZB, and ADH; resources, TK and IK; data curation, TK, IK, AC, and AH; writing, AH, TK, AC, and IK; supervision, OV and IK; funding acquisition, OV, IK, AC, and TK. All authors have read and agreed to the published version of the manuscript.
