# Supplementary figures and images for: Effects of genetic components of plant development on yield-related traits in wheat (Triticum aestivum L.) under stress-free conditions
Source: Front Plant Sci. 2023 Feb 8;13:1070410. doi: 10.3389/fpls.2022.1070410 (PMC9945125; doi:10.3389/fpls.2022.1070410)

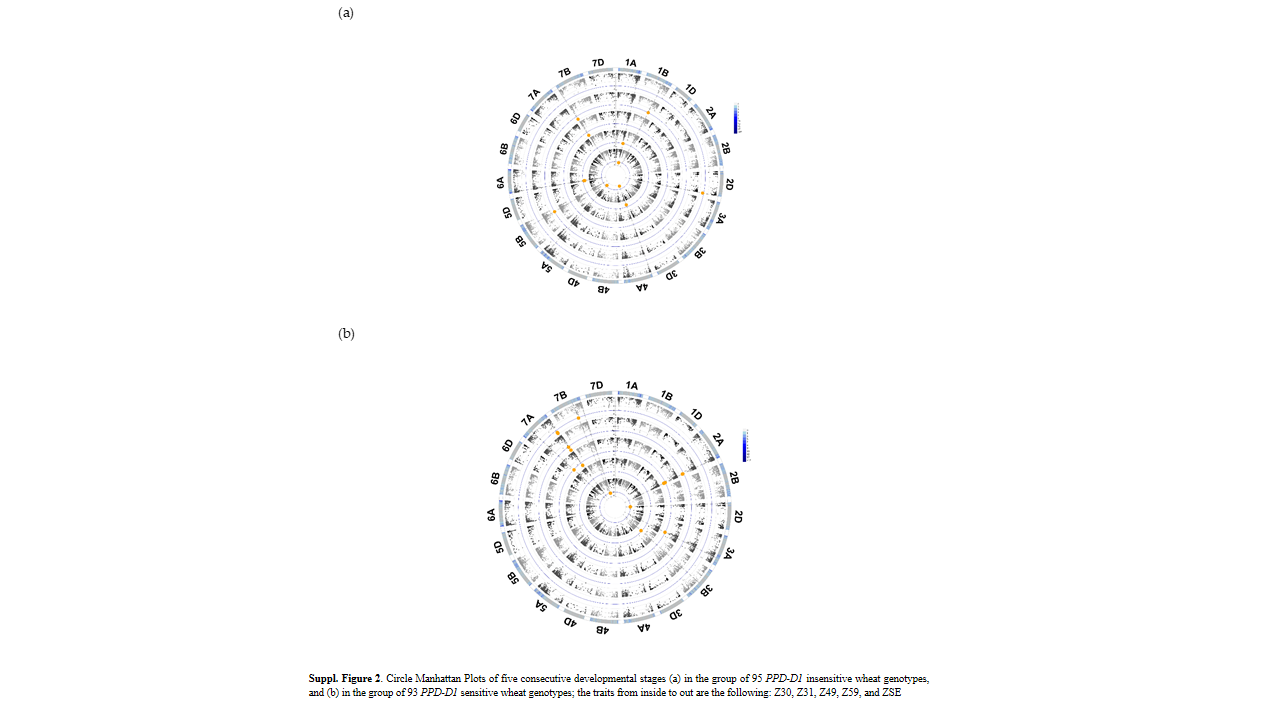

Supplement: Supplementary file 1 [file Presentation_1.zip › Suppl information_final/Suppl Fig 2.tif]

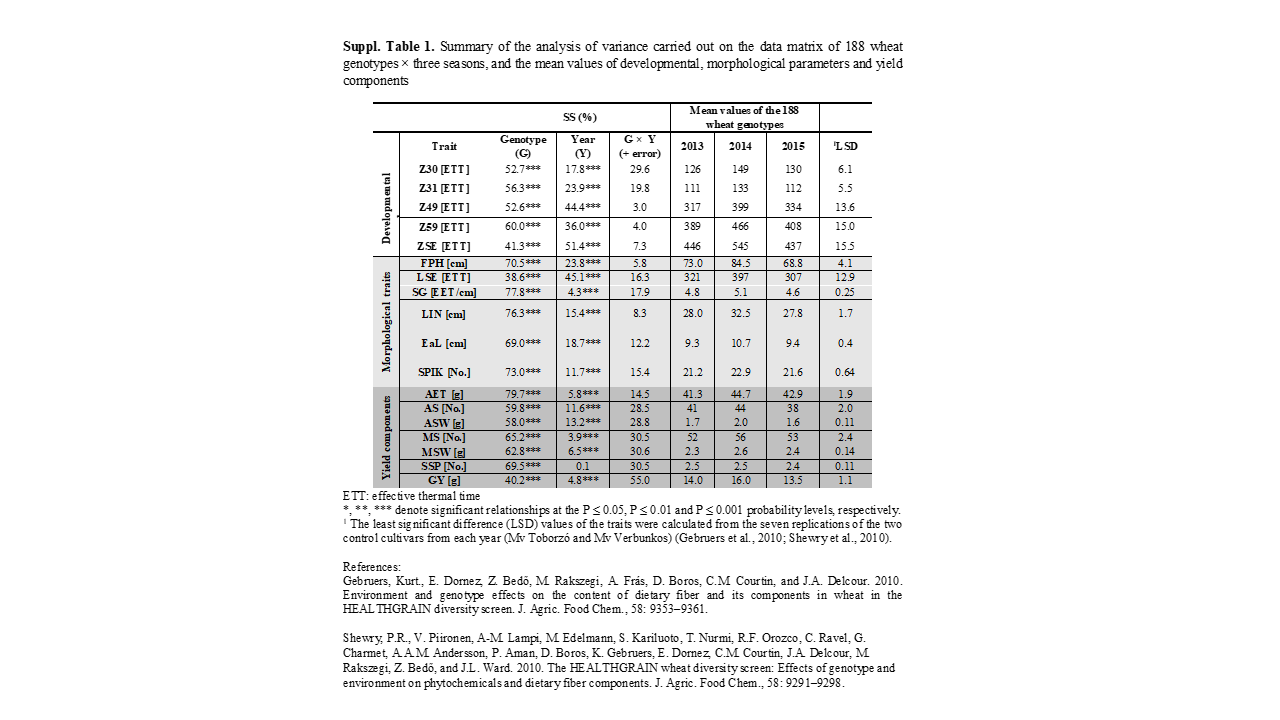

Supplement: Supplementary file 1 [file Presentation_1.zip › Suppl information_final/Suppl Table 1.tif]

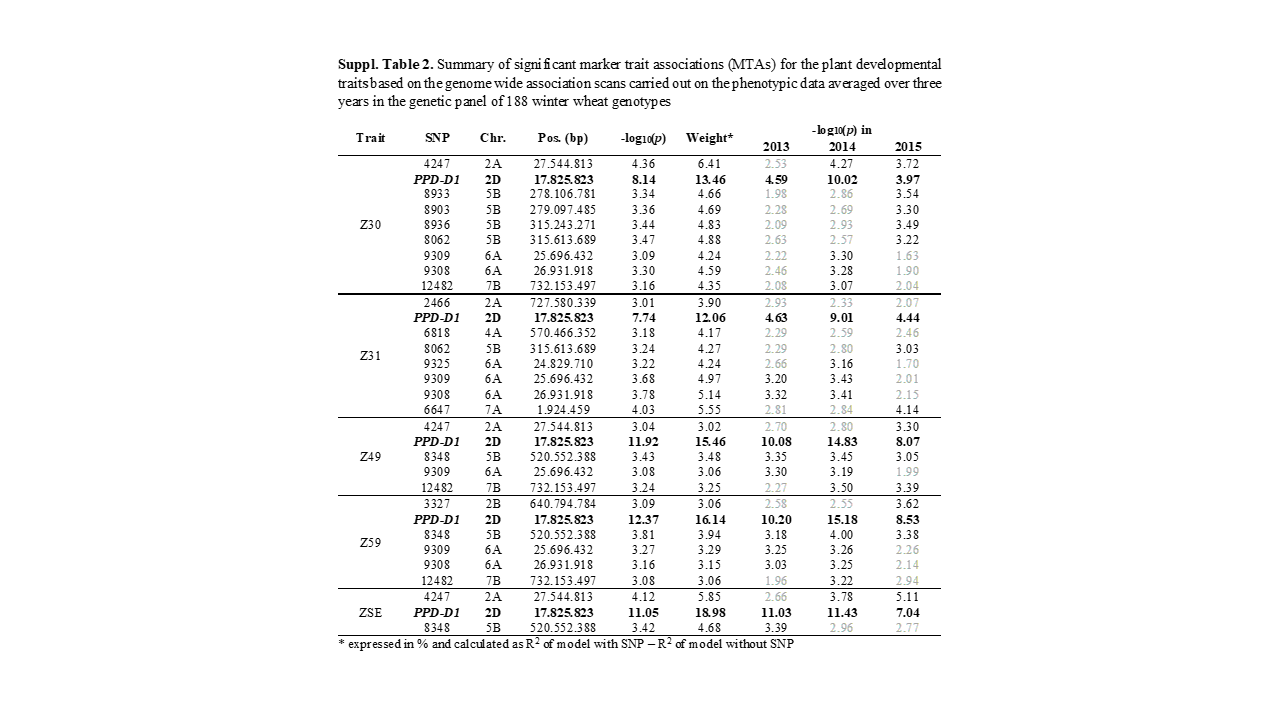

Supplement: Supplementary file 1 [file Presentation_1.zip › Suppl information_final/Suppl Table 2.tif]

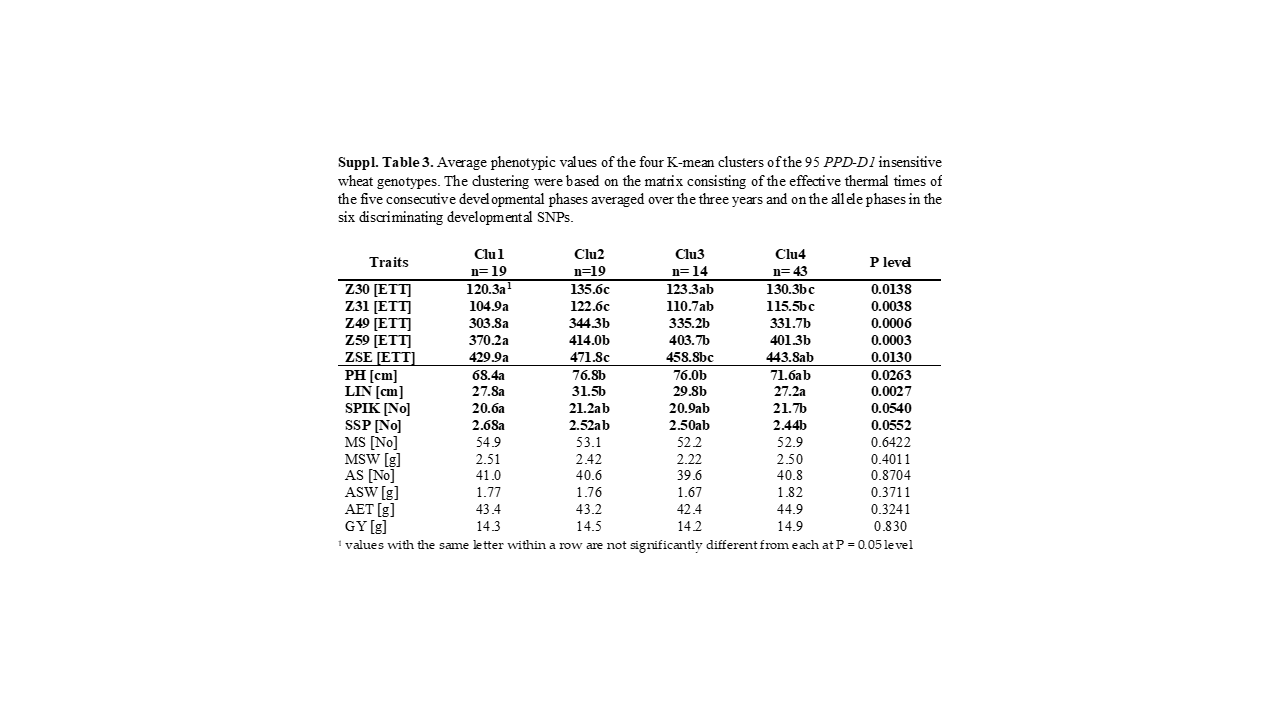

Supplement: Supplementary file 1 [file Presentation_1.zip › Suppl information_final/Suppl Table 3.tif]

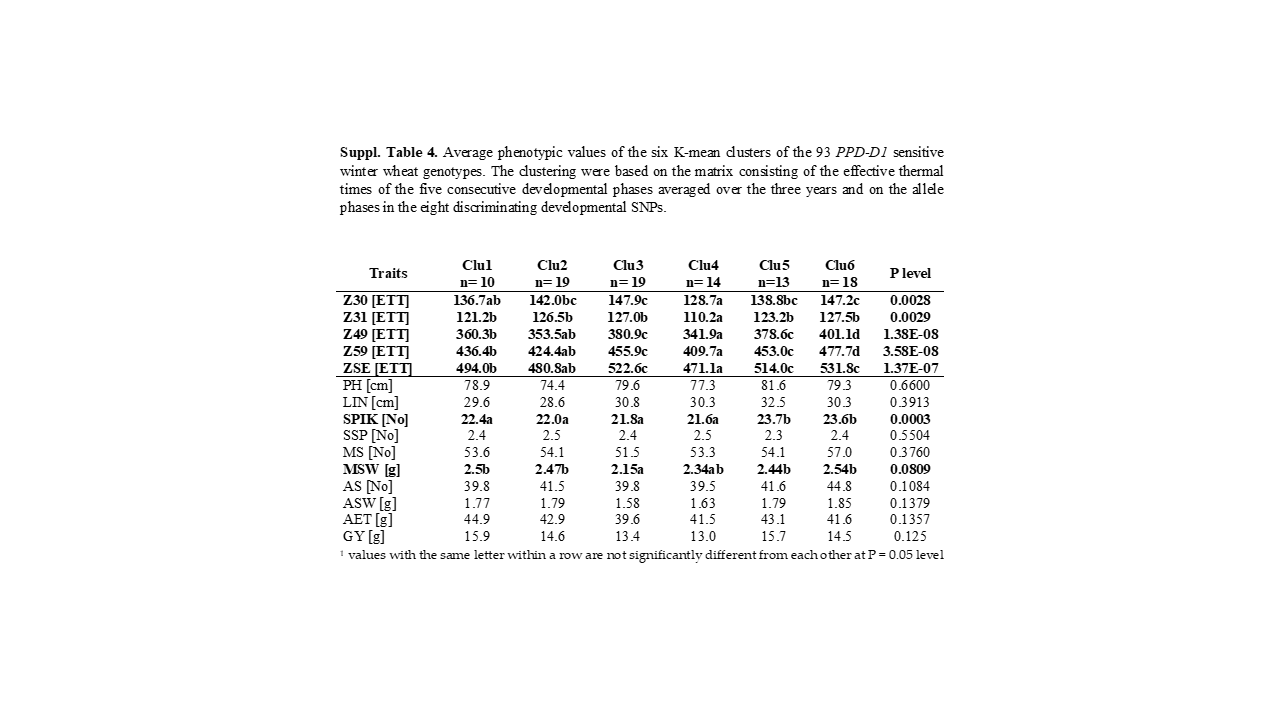

Supplement: Supplementary file 1 [file Presentation_1.zip › Suppl information_final/Suppl Table 4.tif]

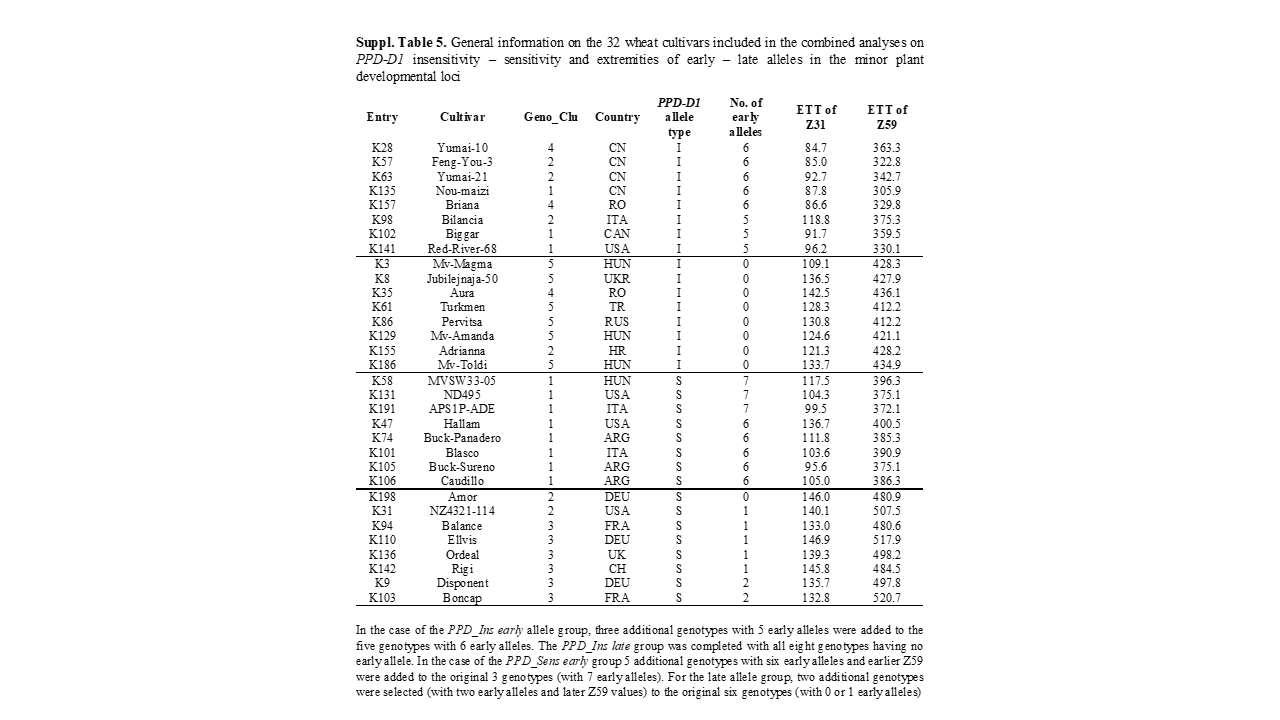

Supplement: Supplementary file 1 [file Presentation_1.zip › Suppl information_final/Suppl Table 5.tif]

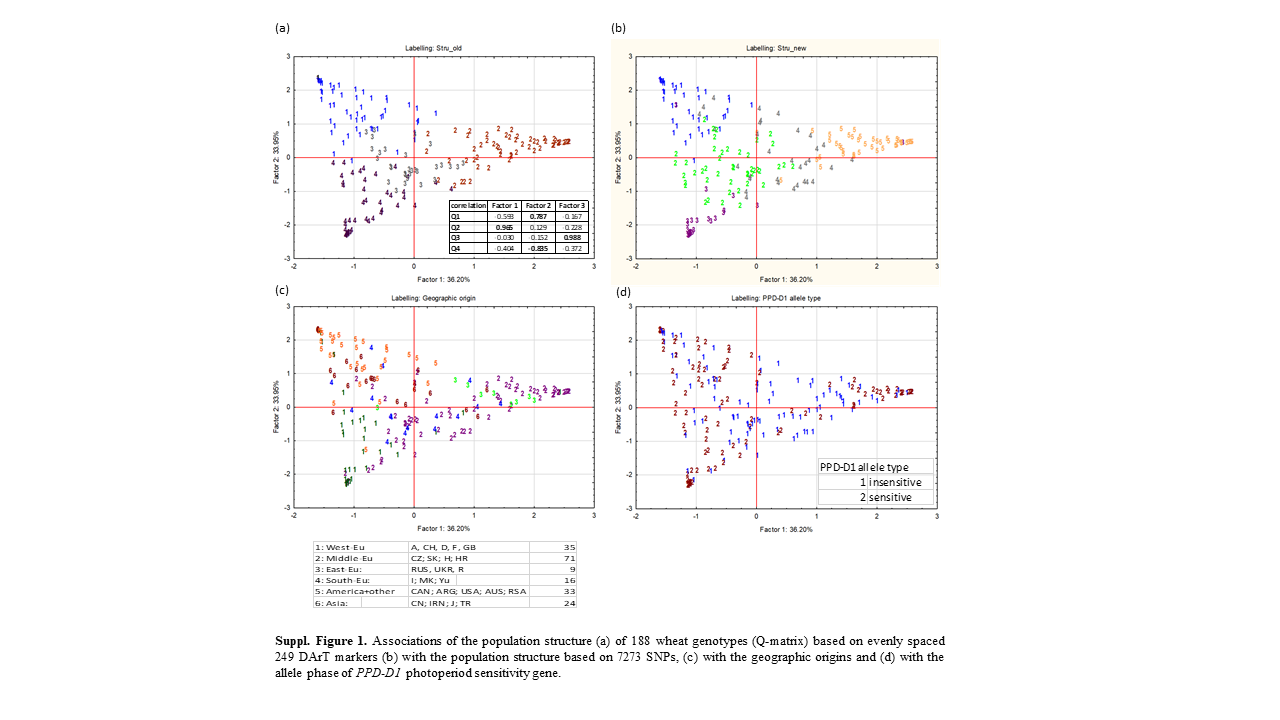

Supplement: Supplementary file 1 [file Presentation_1.zip › Suppl information_final/Suppl Fig 1.tif]
